# Genetic Ablation of Prorenin Receptor in the Rostral Ventrolateral Medulla Influences Blood Pressure and Hydromineral Balance in Deoxycorticosterone-Salt Hypertension

**DOI:** 10.1093/function/zqad043

**Published:** 2023-08-07

**Authors:** Natalia M Mathieu, Eva M Fekete, Patricia C Muskus, Daniel T Brozoski, Ko-Ting Lu, Kelsey K Wackman, Javier Gomez, Shi Fang, John J Reho, Connie C Grobe, Ibrahim Vazirabad, Gary C Mouradian, Matthew R Hodges, Jeffrey L Segar, Justin L Grobe, Curt D Sigmund, Pablo Nakagawa

**Affiliations:** Department of Physiology, Medical College of Wisconsin, Milwaukee, WI 53226, USA; Department of Physiology, Medical College of Wisconsin, Milwaukee, WI 53226, USA; Department of Physiology, Medical College of Wisconsin, Milwaukee, WI 53226, USA; Department of Physiology, Medical College of Wisconsin, Milwaukee, WI 53226, USA; Department of Physiology, Medical College of Wisconsin, Milwaukee, WI 53226, USA; Department of Physiology, Medical College of Wisconsin, Milwaukee, WI 53226, USA; Department of Physiology, Medical College of Wisconsin, Milwaukee, WI 53226, USA; Department of Physiology, Medical College of Wisconsin, Milwaukee, WI 53226, USA; Department of Physiology, Medical College of Wisconsin, Milwaukee, WI 53226, USA; Comprehensive Rodent Metabolic Phenotyping Core, Medical College of Wisconsin, Milwaukee, WI 53226, USA; Department of Pediatrics, Medical College of Wisconsin, Milwaukee, WI 53226, USA; Department of Physiology, Medical College of Wisconsin, Milwaukee, WI 53226, USA; Department of Physiology, Medical College of Wisconsin, Milwaukee, WI 53226, USA; Cardiovascular Center, Medical College of Wisconsin, Milwaukee, WI 53226, USA; Neuroscience Research Center, Medical College of Wisconsin, Milwaukee, WI 53226, USA; Department of Physiology, Medical College of Wisconsin, Milwaukee, WI 53226, USA; Neuroscience Research Center, Medical College of Wisconsin, Milwaukee, WI 53226, USA; Department of Pediatrics, Medical College of Wisconsin, Milwaukee, WI 53226, USA; Department of Physiology, Medical College of Wisconsin, Milwaukee, WI 53226, USA; Comprehensive Rodent Metabolic Phenotyping Core, Medical College of Wisconsin, Milwaukee, WI 53226, USA; Cardiovascular Center, Medical College of Wisconsin, Milwaukee, WI 53226, USA; Neuroscience Research Center, Medical College of Wisconsin, Milwaukee, WI 53226, USA; Department of Biomedical Engineering, Medical College of Wisconsin, Milwaukee, WI 53226, USA; Department of Physiology, Medical College of Wisconsin, Milwaukee, WI 53226, USA; Cardiovascular Center, Medical College of Wisconsin, Milwaukee, WI 53226, USA; Neuroscience Research Center, Medical College of Wisconsin, Milwaukee, WI 53226, USA; Department of Physiology, Medical College of Wisconsin, Milwaukee, WI 53226, USA; Cardiovascular Center, Medical College of Wisconsin, Milwaukee, WI 53226, USA; Neuroscience Research Center, Medical College of Wisconsin, Milwaukee, WI 53226, USA

**Keywords:** hypertension, blood pressure, autonomic function, rostral ventrolateral medulla, prorenin receptor, Atp6ap2, renin–angiotensin system, DOCA-salt hypertension, sympathetic nervous system, sex-difference

## Abstract

Non-enzymatic activation of renin via its interaction with prorenin receptor (PRR) has been proposed as a key mechanism of local renin–angiotensin system (RAS) activation. The presence of renin and angiotensinogen has been reported in the rostral ventrolateral medulla (RVLM). Overactivation of bulbospinal neurons in the RVLM is linked to hypertension (HTN). Previous studies have shown that the brain RAS plays a role in the pathogenesis of the deoxycorticosterone (DOCA)-salt HTN model. Thus, we hypothesized that PRR in the RVLM is involved in the local activation of the RAS, facilitating the development of DOCA-salt HTN. Selective PRR ablation targeting the RVLM (PRR^RVLM-Null^ mice) resulted in an unexpected sex-dependent and biphasic phenotype in DOCA-salt HTN. That is, PRR^RVLM-Null^ females (but not males) exhibited a significant delay in achieving maximal pressor responses during the initial stage of DOCA-salt HTN. Female PRR^RVLM-Null^ subsequently showed exacerbated DOCA-salt-induced pressor responses during the “maintenance” phase with a maximal peak at 13 d on DOCA-salt. This exacerbated response was associated with an increased sympathetic drive to the resistance arterioles and the kidney, exacerbated fluid and sodium intake and output in response to DOCA-salt, and induced mobilization of fluids from the intracellular to extracellular space concomitant with elevated vasopressin. Ablation of PRR suppressed genes involved in RAS activation and catecholamine synthesis in the RVLM but also induced expression of genes involved in inflammatory responses. This study illustrates complex and sex-dependent roles of PRR in the neural control of BP and hydromineral balance through autonomic and neuroendocrine systems.

Graphical abstract

## Graphical Abstract

**Graphical Abstract:**
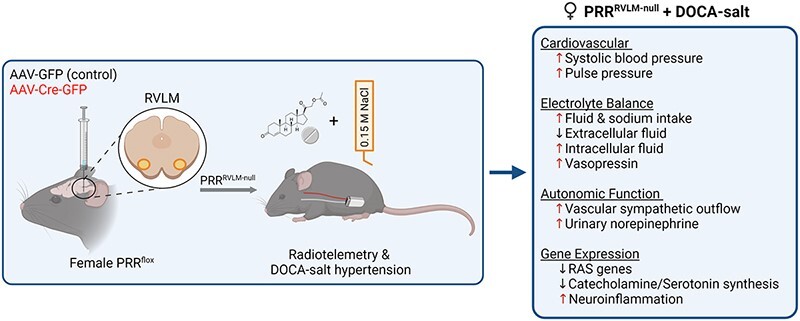

## Introduction

Hypertension (HTN) is considered one of the most important health issues of the last few decades due to its high incidence and serious complications. According to the World Health Organization and the Centers for Disease Control and Prevention, HTN affects more than 1.3 billion people worldwide, including more than 100 million adults in the United States.^1,2^ Importantly, HTN is responsible for 85 million deaths worldwide from heart disease, stroke, and kidney disease. Despite the wide variety of anti-hypertensive drugs available, there are still a large number of patients that are refractory to these conventional therapies.^3,4^ The renin–angiotensin system (RAS) is one of the most studied mechanisms of blood pressure (BP) regulation and an important pharmacological target widely used in clinical settings. Renin is the rate-limiting enzyme for angiotensin (ANG)-II production, the primary bioactive product of the RAS. However, only 15% of patients with essential HTN exhibit increased plasma renin activity.^5^ Indeed, approximately 60% of patients with resistant HTN exhibit low plasma renin.^6^

In addition to the classical circulating RAS, the tissue-specific RAS operates locally in the brain, heart, vasculature, adrenal gland, reproductive tract, kidneys, and many other organs (reviewed in^7^). The existence of the brain RAS and its importance in BP regulation, hydromineral balance, autonomic function, and metabolism is well accepted.^8–11^ Dysregulation of the brain RAS plays a key mechanistic role in the development and maintenance of high BP and energy balance in models exhibiting low plasma renin, such as DOCA-salt HTN.^12^ This implies the existence of de novo generation of ANG-II within brain regions implicated in cardiovascular and metabolic control. Angiotensinogen (AGT), the only known renin substrate, is constitutively expressed in astrocytes and some neurons.^13^ Indeed, the brain’s extracellular space is “bathed” in AGT. However, it remains controversial whether renin, the key limiting enzyme of the ANG-II biosynthetic cascade, is necessary for the cleavage of AGT to generate ANG-I in the brain.^14,15^ In the kidney, renin is translated as an inactive proenzyme (prorenin) that requires hydrolytic cleavage of its prosegment to attain its full enzymatic activity. Intriguingly, evidence suggests that the enzymes responsible for the hydrolytic cleavage of prorenin may be absent in the brain.^16,17^ In the last decade, several studies have demonstrated that prorenin receptor (PRR), a transmembrane protein encoded by *Atp6ap2*, has the capacity to bind prorenin and induce a non-enzymatic activation of renin without prosegment cleavage. Studies have reported that expression of PRR in different regions of the brain participates in the regulation of BP and fluid balance through RAS-dependent mechanisms, and lack of PRR prevents the development of HTN in several preclinical models.^18–20^ However, recent studies have identified many other functions of PRR unrelated to the regulation of the RAS. For example, PRR is implicated in cellular processes, such as endosomal trafficking, autophagy, neuroinflammation, and RAS-independent signal transduction, that involve different mitogen-activated protein kinases.^21–24^ Thus, whether PRR mediates its effects through the activation of the RAS remains elusive.

Our laboratory has previously identified the existence of renin-expressing neurons in close proximity to AGT-expressing cells within the rostral ventrolateral medulla (RVLM) region.^25^ This finding was not surprising as the RVLM is a region of the brainstem that plays a critical role in regulating cardiovascular function and BP via the sympathetic nervous system. The RVLM has been implicated in several cardiovascular disorders, including HTN, heart failure, and arrhythmia.^26^ Thus, we hypothesized that expression of PRR in the RVLM is required for the full development and maintenance of HTN through activation of the brain RAS. To test the physiological impact of PRR in the RVLM, comprehensive cardiovascular, renal, and metabolic studies were conducted in mice lacking PRR in the RVLM during DOCA-salt HTN. This study demonstrates a complex sex-specific role of PRR in the RVLM during the development and maintenance of DOCA-salt HTN.

## Methods

The protocols for acute studies, neuronal cell culture experiments, telemetry recordings, power spectral analyses, biochemical assays, in situ hybridization, histological studies, acute saline challenge, transcutaneous glomerular filtration rate (tGFR), non-invasive pulse-wave velocity (PWV), nuclear magnetic resonance (NMR), bioimpedance spectroscopy (BIS), western blotting, and bulk RNA sequencing are described in the Supplemental Online Methods.

### Animal Subjects

This study was conducted using C57BL/6J mice (Stock 000664, Jackson Laboratories, ME) and mice carrying floxed *Atp6ap2* encoding PRR, which was generously provided by Frederique Yiannikouris (University of Kentucky) with permission from Genevieve Nguyen, who developed the mouse model.^27,28^ Both males and females were 12–14 wk of age at the onset of the experiments. Mice were provided standard laboratory chow (Envigo/Teklad #2920, IN), and chlorinated tap water was available *ad libitum* during the experimental procedures. All animals were housed at standard room temperature (∼22°C) under a 14:10 light–dark cycle (light onset at 5 am). Surgical and experimental procedures adhered to the National Institutes of Health’s “Guide for the Care and Use of Laboratory Animals” and were approved by the Medical College of Wisconsin and the University of Iowa Animal Care and Use Committees.

### RVLM-Targeted Microinjection

Radiotelemetry-instrumented mice were anesthetized with isoflurane inhalation (1.5–2.0%) and securely placed in a stereotaxic frame. The RVLM coordinates were calculated using the Paxinos and Franklin mouse brain atlas: 1.25 mm mediolateral, 1.8 mm caudal to lambda, and 6.4 mm ventral from the dorsal surface of the skull.^29^ Once the coordinate were localized, a 1.0 mm diameter hole was made using a hand drill to penetrate the skull and expose the brain. A 32-gauge Neuros syringe (Hamilton, NV) was used to deliver drugs or viruses. Before treatments, the correct placement of the injector was confirmed by observing a pressor response to L-glutamate (Sigma Aldrich, MO).

### Conditional Ablation of PRR in the RVLM

Homozygous females and hemizygous males carrying exon 2-floxed PRR allele in the X chromosome (PRR^f/f^ and PRR^f/y^, respectively), with or without a tdTomato Cre reporter allele (ROSA) were used. First, to validate the animal model, we bred PRR^f/y^ with Ai14, tdTomato Cre reporter line (Stock #007914, Jackson Laboratories). These mice were subjected to stereotactic bilateral microinjections of either adenovirus (ad)-green fluorescent protein (GFP) or Ad-CRE recombinase fused with GFP using the coordinates described above. After 2 wk, the targeted region was dissected under a fluorescent stereo microscope. Subsequently, DNA was extracted from one-half of the collected tissues using the PicoPure DNA Extraction Kit (catalog # KIT0103, Applied Biosystems) and subjected to PCR validation. Excision of exon 2 was confirmed using PCR amplification with the following set of primers: 5′-AGCACTCTCTTCCAGGTATGTTGTG-3′ and 5′-GCCCCTCTCTTACAGTTCTATCAGT-3′. The presence of a 326 bp product indicated excision of PRR. Cycling conditions were the following: 94°C for 3 minutes; 2 cycles of 94°C for 1 minute, 62°C for 1 minute, 72°C for 1 minute; 30 cycles of 94°C for 30 s, 62°C for 30 s, 72°C for 30 s, and 1 cycle of 72°C for 1 minute. The other half of the tissues were homogenized with RIPA buffer containing phenylmethylsulfonyl fluoride, sodium orthovanadate, and proteinase inhibitors (Catalog # sc-24948, Santa Cruz Biotechnology, TX), sonicated, and incubated at 4°C for 30 minutes. The homogenate was centrifuged at 13 000* g* for 10 minutes, and the supernatant was stored at –80°C until protein analysis by western blotting, as described in the supplemental methods section.

In another set of animals, the RVLM of PRR^f/y^ was targeted with either adeno-associated virus (AAV) 2/2-CMV-GFP or AAV2/2-CMV-CRE-GFP, and brain punches from the RVLM were collected. These tissues were subjected to total RNA extraction using TRIzol reagent (Thermo Fisher Scientific, NA), followed by reverse transcription using the Superscript III reverse transcriptase kit (Invitrogen, CA). To detect PRR (*Atp6ap2*) and 18s (housekeeping gene), a real-time PCR was performed using the Fast SYBR Green Master Mix (Invitrogen, CA) and the following primer set: *Atp6ap2*: 5′-TCTCTCCGAACTGCAAGTGCAACA-3′ and 5′-CCAAACCTGCCAGCTCCAATGAAT-3′; *18s*: 5′-CGCTTCCTTACCTGGTTGAT-3′ and 5′-GAGCGACCAAAGGAACCATA-3′, respectively.

### Chronic Studies in DOCA-Salt Model

Male and female 12–14-wk-old PRR^flox^ mice or wildtype (WT) littermates were divided into 4 groups: (a) male PRR^f/y^ + AAV-GFP (male WT), (b) male PRR^f/y^ + AAV-CRE-GFP (male PRR^RVLM-Null^), (c) female PRR^f/f^ + AAV-GFP (female WT), and (d) female PRR^f/f^ + AAV-CRE-GFP (female PRR^RVLM-Null^). The first cohort was instrumented with radiotelemetric devices for BP recordings, as described in the supplemental section. After 48 hours, viruses were delivered bilaterally into the RVLM as aforementioned. Pressor response to a small dose of L-glutamate at the injection site was used to confirm the correct placement of the injector in the RVLM. At least 3 wk were allowed to induce expression of Cre. After a 3-d baseline recording, DOCA-salt HTN was induced by a subcutaneous implant of a 50 mg DOCA pellet (Deoxycorticosterone acetate, Cat #D7000, Millipore-Sigma, MO), after which 0.15 M NaCl saline (dissolved in deionized water) was offered as the only source of drinking water. BP, heart rate (HR), and activity were recorded for 21 d. Power spectral analysis of HR variability and diastolic BP variability was performed at baseline, day 3 (early stage), and day 13 (late stage) using HemoLab software as previously described.^30,31^

A second set of animals was subjected to comprehensive metabolic and hydromineral balance phenotyping. Mice were single-housed in metabolic cages (item #52–6756, Hatteras Instruments model MMC100, Harvard Apparatus, MA) to assess food and fluid intake and for urine and feces collection at baseline and days 3, 6, and 13 on the DOCA-salt regimen. Twenty-four-hour urine samples were processed to evaluate urine electrolytes, catecholamines, aldosterone, and copeptin. The urine sodium and potassium concentrations were measured using a flame photometer (Jenway PFP7 or a BWB XP). For norepinephrine/epinephrine, aldosterone, and copeptin measurements, commercially available ELISA kits from Abnova (catalog # KA1877, Taiwan), Cayman Chemicals (catalog # 501090, MI), and Cloud-Clone Corp (catalog # CEA365Mu, PRC) were used, respectively.

### Statistical Analysis

All data are presented as mean ± SEM. The data were analyzed using GraphPad Prism software (Version 9.5.0, Boston, MA). Two-way ANOVA or mixed-effects model with repeated measures as appropriate, followed by Sidak’s multiple comparison procedures or a 2-tailed unpaired *t*-test was performed. Survival rate curves were analyzed using the Logrank comparison test and the pairwise comparison of individual survival curves using the Kaplan–Meier method. Outliers were identified by either the interquartile range method and the ROUT method with *Q* = 5%. A *P-value* of less than 0.05 was considered significant. Individual data points were plotted in dot/whisker plots if appropriate. In all experiments, animals were randomly assigned into two experimental groups, either WT or PRR^RVLM-Null^.

## Results

### PPR is Expressed in the Brainstem and is Implicated in BP Control

**Figure 1.**
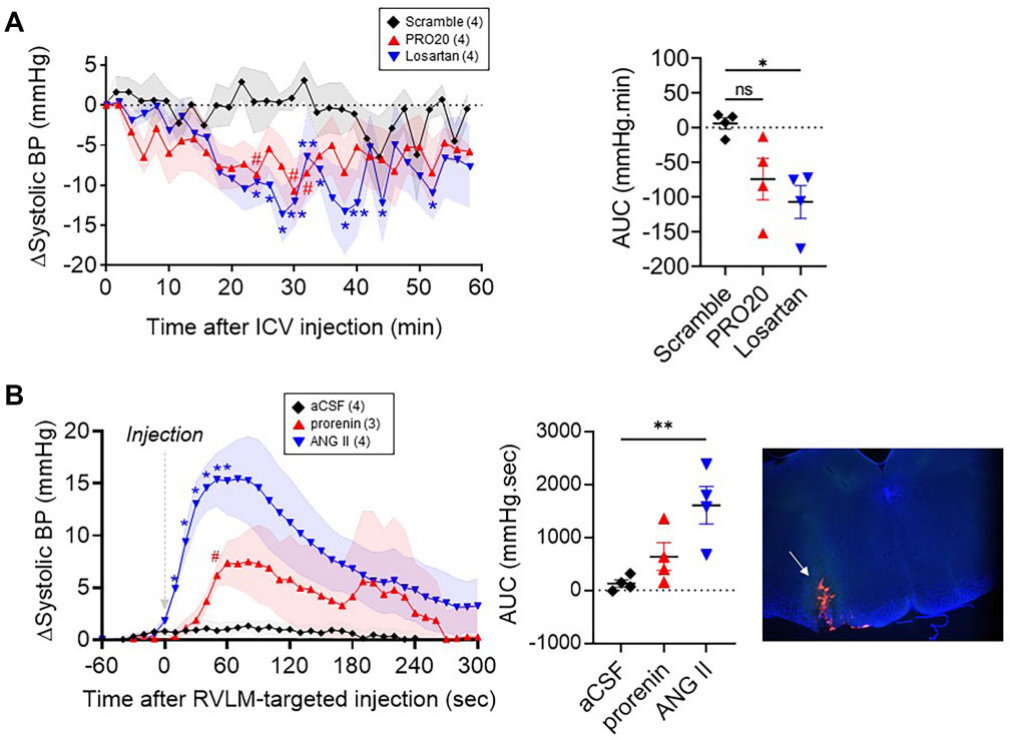
(A) The systolic BP change following acute intracerebroventricular infusion of PRR antagonist (PRO20), losartan, and control scramble peptide in C57BL/6J mice under deoxycorticosterone-acetate (DOCA)-salt-induced hypertension. Systolic BP was measured at baseline and during DOCA-salt administration. The change in systolic BP (from baseline, ΔSystolic BP) to ICV drug infusion was analyzed by repeated measures two-way ANOVA with Dunnett multiple comparison *post-hoc* test. Time *P* < .01, Drug *P* = .12, and TxD *P* = .16. The area under the curve (AUC) of ΔSystolic BP was calculated and analyzed by one-way ANOVA followed by the Tukey *post-hoc* test. One-way ANOVA *P* = 0.02, * *P* < .01 losartan versus scramble, ** *P* < .01 losartan versus scramble, and # *P* < .01 PRO20 versus scramble. (B) Acute unilateral injection of artificial cerebrospinal fluid (aCSF), recombinant prorenin, or angiotensin II (ANG II) targeting the RVLM. The ΔSystolic BP was analyzed by repeated measures two-way ANOVA with Dunnett multiple comparison *post-hoc* test. Time *P* = .01, Drug *P* = .15, TxD *P* < .01. The AUC was analyzed by one-way ANOVA followed by Tukey *post-hoc* test. One-way ANOVA *P* < .01, *P < .01 ANG II versus aCSF, and # *P* < .01 recombinant prorenin versus aCSF. After the treatments, fluorescent microspheres were delivered, and the brains were sectioned to confirm the correct placement of the injector in the RVLM region with epi-fluorescent microscopy.

**Figure 2.**
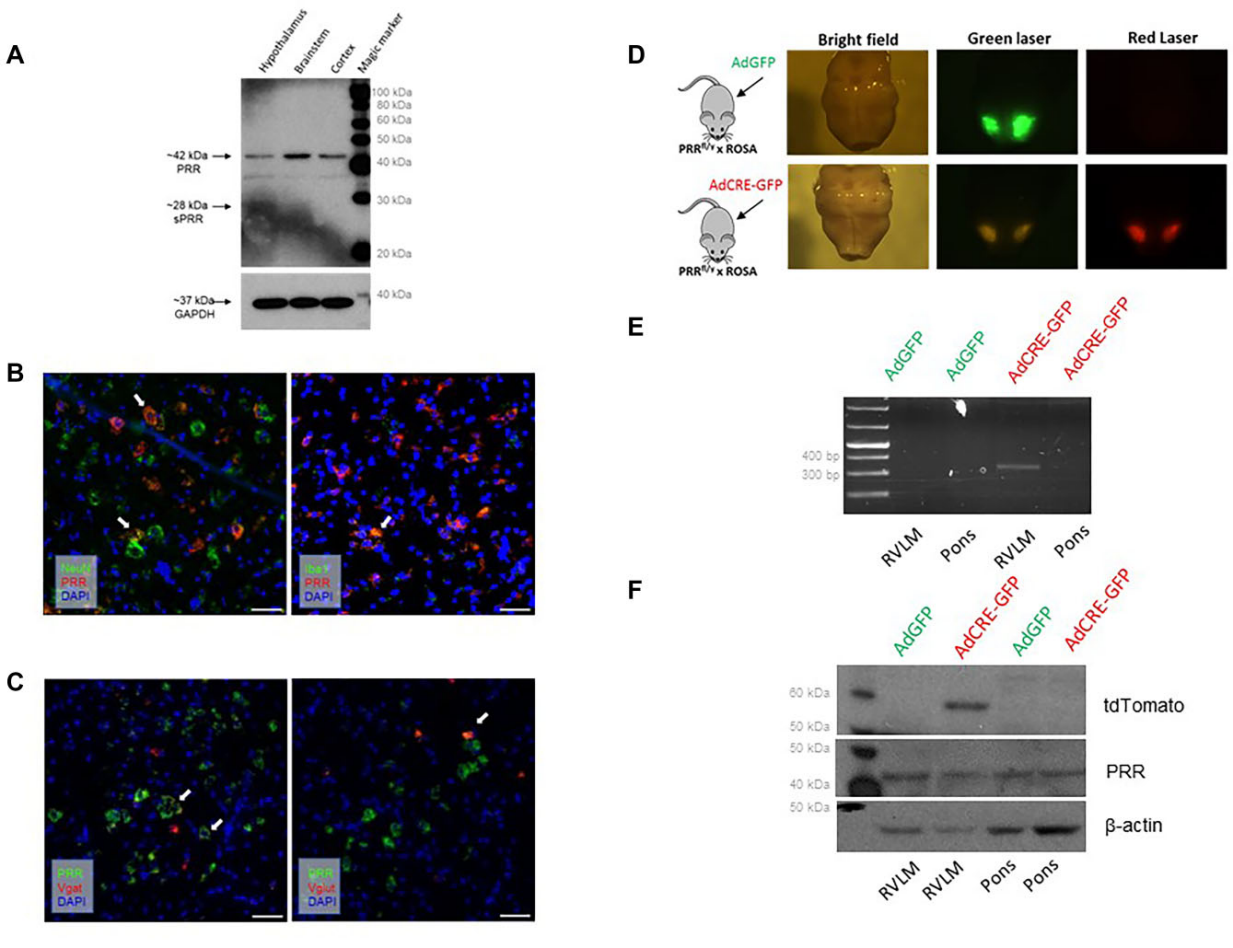
(A) Expression of PRR in tissue homogenates of the hypothalamus, brainstem, and cortex was detected by western blotting on C57BL/6J mice. (B) PRR (red) is located on both in neurons (NeuN, green, left panel) and microglia (iba1, green, right panel) in the RVLM. (C) PRR (green) colocalized with vesicular gamma-aminobutyric acid (GABA)-positive inhibitory neurons (slc32a1, red) but also vesicular glutamate transporter 2 (Vglut)-positive exhitatory neurons (slc17a6, red) in the RVLM. Colocalization is indicated with white arrows, respectively. Scale bar = 50 μM. (D) Genetic ablation of PRR in the RVLM in PRR-flox mice: Representative image of the brainstem in bright field, green and red laser excitation. Mice carrying exon 2-floxed PRR allele (PRR^f/y^) and a tdTomato Cre reporter allele (ROSA) were subjected to stereotactic bilateral microinjections of either adenovirus (ad)-green fluorescent protein (GFP; AdGFP) or Ad-Cre recombinase GFP (AdCRE eGFP). The RVLM region was dissected for genomic DNA amplification and protein analysis. (E) DNA was extracted, and PCR was performed using a primer set that amplifies a 326 bp segment indicating excision of exon 2 in PRR gene (*Atp6p2*). A representative PCR agarose gel is shown. A 326 bp band appears in the RVLM of mice microinjected in the RVLM with AdCRE-GFP but not for the Ad-GFP control. (F) Protein expression of tdTomato (top) and PRR (bottom).

To confirm the importance of PRR in the neurogenic control of BP, we evaluated whether pharmacological blockade of PRR in the brain ameliorates established HTN in C57BL/6J mice subjected to DOCA-salt HTN. Similar to what was reported by Li et al.^32^, intracerebroventricular (ICV) infusion of PRR antagonist (PRO20) elicited an acute reduction in systolic BP comparable to ICV-losartan (Figure 1A). Given the premises that: (1) administration of ANG-II in the RVLM elicits pressor and autonomic responses^33^ and (2) neurons with renin promoter activity are localized in close proximity to AGT-expressing cells in the RVLM^25^, we hypothesized that PRR contributes to local RAS activation in this brain region. First, we performed acute microinjections of recombinant prorenin targeting the RVLM in anesthetized mice. Vehicle and ANG-II were used as negative and positive controls, respectively. RVLM-targeted administration of recombinant prorenin elicited a transient 7.5 ± 3.1 mmHg increase in systolic BP, which peaked at 80 s post-injection. In contrast, ANG-II elicited a 16.8 ± 3.4 mmHg increase, which peaked at 50 s post-injection. Vehicle did not elicit significant changes in BP (Figure 1B). These data confirm that local activation of the RAS in the RVLM is sufficient to increase BP and also suggest that the RVLM likely contains the machinery to activate prorenin. Furthermore, administration of recombinant prorenin in cultured brainstem neurons elicited intracellular signaling activation evidenced by elevated phosphorylated extracellular signal-regulated kinase 1/2 (ERK) normalized by total ERK (Figure S1A) and increased superoxide production (Figure S1B). Protein analysis of brain homogenates confirmed that PRR is expressed in the hypothalamus, brainstem, and cortex (Figure 2A). Furthermore, the multicolor in situ mRNA hybridization technique revealed that PRR is expressed in neuronal and microglial cell populations within the RVLM (Figure 2B). We also observed that PRR is expressed in both inhibitory GABAergic- and excitatory glutaminergic neurons (Figure 2C).

### Genetic Ablation of PRR Targeting the RVLM Results in Biphasic and Sex-Dependent Phenotype in Response to DOCA-Salt

**Figure 3.**
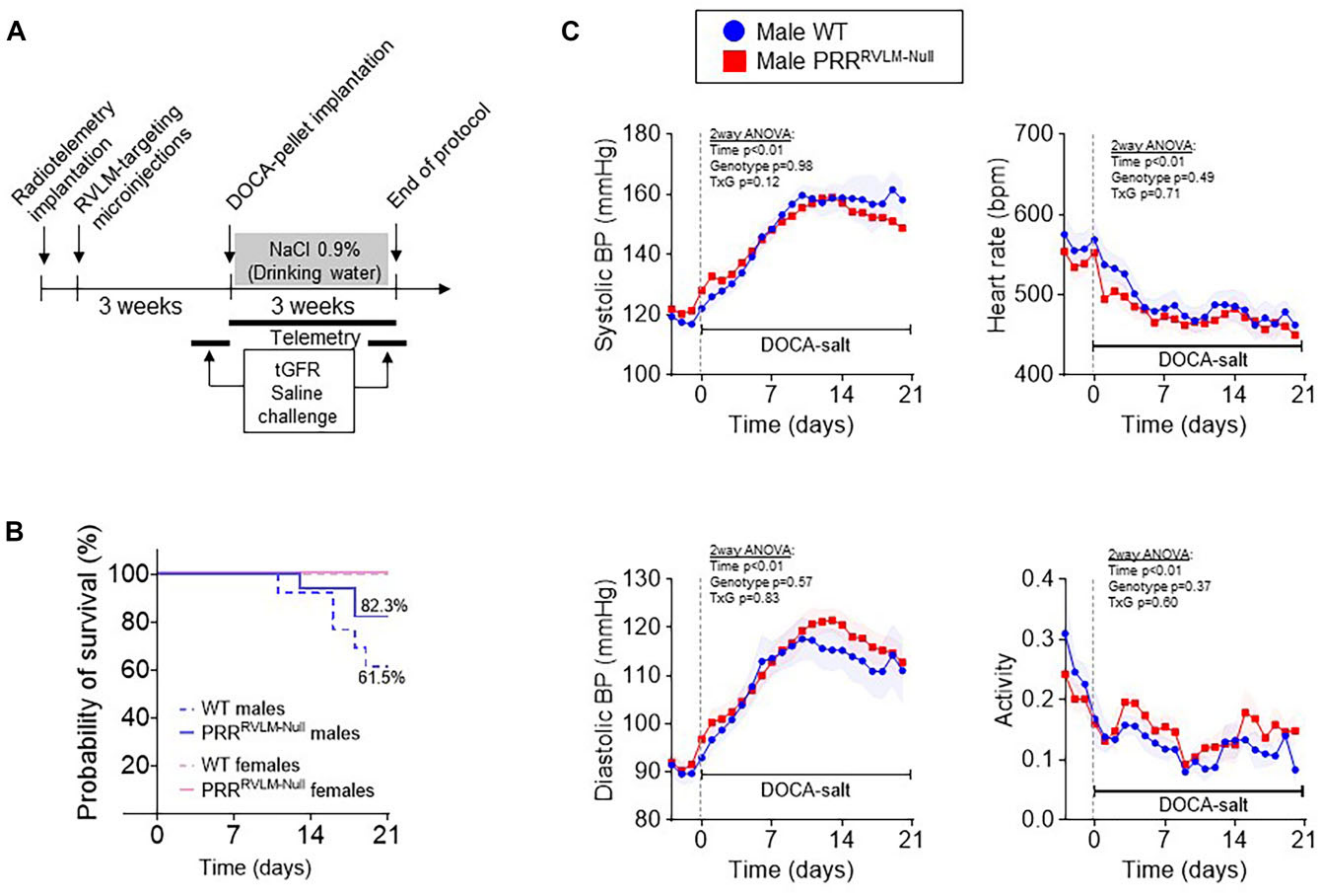
(A) Schematic representation of the experimental design to evaluate cardiovascular, metabolic, and renal phenotypes in mice lacking PRR in the RVLM region under normal and deoxycorticosterone-acetate (DOCA)-salt-induced hypertension. (B) Survival rate in male and female WT and PRR^RVLM-Null^ upon DOCA-salt administration. Survival rate curves were analyzed using the Logrank comparison test and the pairwise comparison of individual survival curves using the Kaplan–Meier method. Logrank test *P* < .01, Kaplan–Meier test WT versus PRR^RVLM-KO^ males *P* = .2, Kaplan–Meier test WT versus PRR^RVLM-KO^ females *P* > .99. (C) Cardiovascular effects of PRR deletion in the RVLM region in males. Male PRR-flox mice were subjected to RVLM-targeted microinjection of AAV-CRE GFP (PRR^RVLM-Null^, *n* = 15) or control virus AAV-GFP (WT, *n* = 8). Daily average systolic BP, diastolic BP, HR, and activity during baseline and DOCA-salt. Data are expressed as mean ± SEM and were analyzed by repeated measures 2-way ANOVA followed by Sidak’s multiple comparison *post*-*hoc* test. Transcutaneous glomerular filtration rate (tGFR).

To induce conditional ablation of PRR in the RVLM, double transgenic mice carrying floxed *Atp6ap2* encoding PRR and the Cre-activatable tdTomato reporter gene (PRR^flox^ x ROSA) were subjected to RVLM-targeted stereotactic microinjection of adenoviruses to deliver either Cre recombinase and green fluorescent protein (AdCRE-GFP) or a control GFP (AdGFP) (Figure 2D). Microinjection of Ad-CRE-GFP but not Ad-GFP induced: (1) tdTomato expression in the RVLM indicative of Cre recombinase activity (Figure 2D) and (2) recombination and deletion of *Atp6a2* exon 2 restricted to the RVLM evidenced by the presence of the PCR band at 326 bp indicative of Cre-mediated recombination between loxP sites in the RVLM but not in the pons (Figure 2E). Protein analyses of RVLM punches indicated expression of tdTomato and a 33.6% reduction of PRR in the RVLM (Figure 2F). Of note, the brain punch technique might contain non-RVLM tissue. Delivery of Cre recombinase was also effective using an AAV, evidenced by an inability to detect PRR mRNA expression by RT-qPCR in mice subjected to AAV-CRE-GFP (data not shown). Given the effectiveness of AAV infections, all subsequent experiments were designed to evaluate the role of PRR in the RVLM on BP and autonomic control in response to DOCA-salt HTN employed AAV-CRE-GFP or AAV-GFP (Figure 3A).

**Figure 4.**
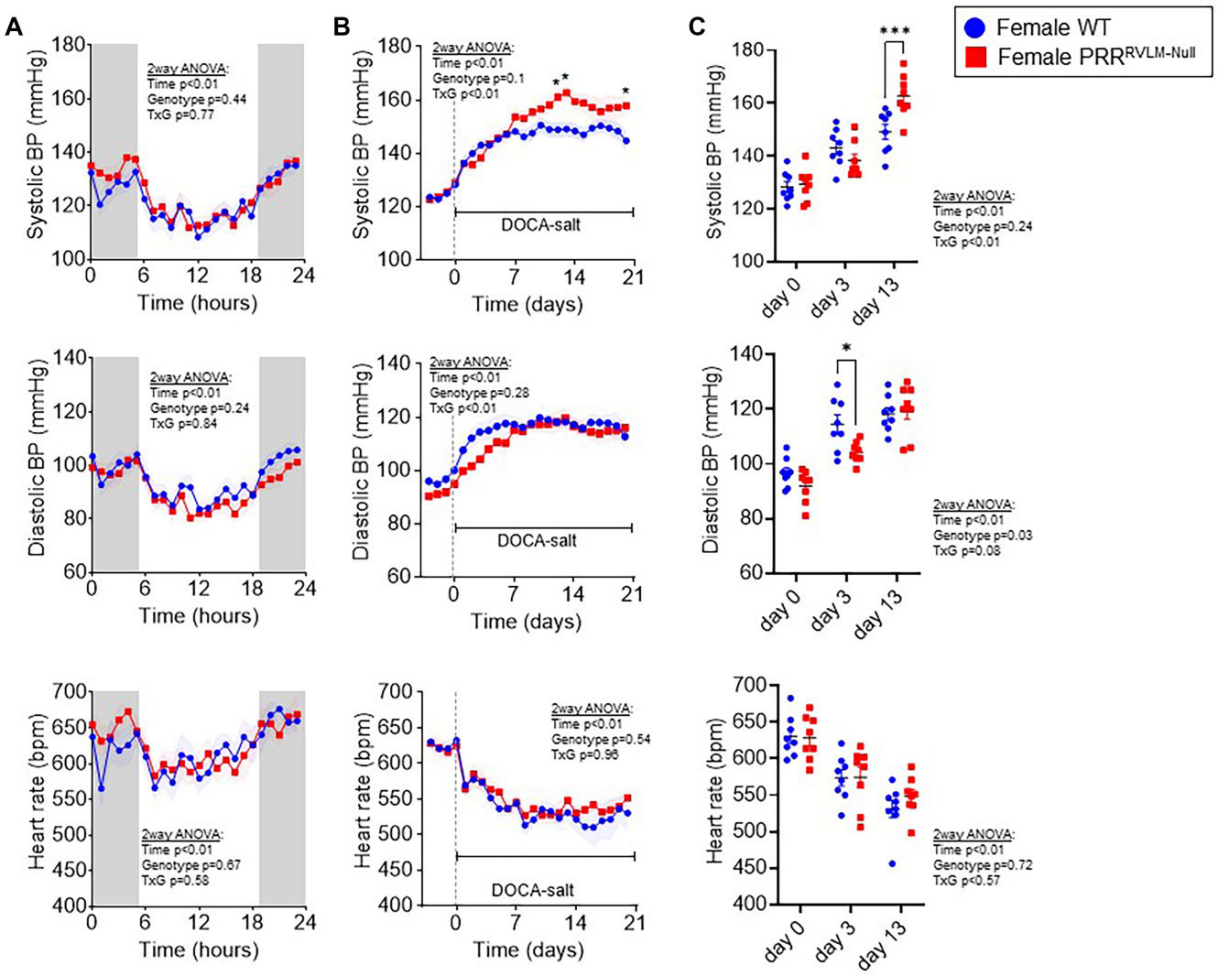
Cardiovascular effects of PRR deletion in the RVLM region in females. Female PRR^f/f^ mice were subjected to RVLM-targeted microinjection of AAV-CRE GFP (PRR^RVLM-Null^, *n* = 8) or control virus AAV-GFP (WT, *n* = 8). (A) Average hourly systolic BP (top), diastolic BP (middle), and HR (bottom) at baseline. Shaded boxes depict dark cycle. (B) Averaged daily systolic BP (top), diastolic BP (middle), and HR (bottom) before and during deoxycorticosterone-acetate (DOCA)-salt administration. (C) Systolic BP (top), diastolic BP (middle), and HR (bottom) of individual animals at 0, 3, and 13 d with DOCA-salt treatment. Data are expressed as mean ± SEM and were analyzed by repeated measures 2-way ANOVA followed by Sidak’s multiple comparison *post-hoc* test. * *P* < .05 and *** *P* < .001 versus WT control.

First, we evaluated the effect of RVLM-targeted ablation of PRR on cardiovascular parameters in males and females at baseline and in response to DOCA-salt HTN. Interestingly, control males (WT) subjected to RVLM-targeted microinjection of AAV-GFP, exhibited a 61.5% survival rate by the end of the 21-d protocol. Males subjected to RVLM AAV-Cre-GFP (PRR^RVLM-Null^) exhibited an 82.3% survival rate. However, the difference in survival rate between groups did not reach statistical significance. In contrast, no mortality was observed in females (Figure 3B). As a result of DOCA-salt treatment, all mice exhibited elevated systolic and diastolic BP concomitant with a decrease in HR. Contrary to our expectations, male PRR^RVLM-Null^ showed no significant change in systolic and diastolic BP, HR, or activity compared to male WT under baseline and subsequent DOCA-salt treatment (Figure 3C). In contrast, although female PRR^RVLM-Null^ did not exhibit significant differences in BP or HR at baseline (Figure 4A), DOCA-salt administration unmasked an interesting dimorphic and sex-dependent phenotype. That is, during the initial stage of DOCA-salt infusion (first 6 d), there was a delay in achieving the maximal diastolic and mean BP response (Figure 4B and C, Figure S2). However, during the late stage (days between 12 and 21 on DOCA-salt), female PRR^RVLM-Null^ exhibited a further increase in systolic BP compared to female WT. There was no difference in HR or activity between these groups. Since only females lacking PRR exhibited a prominent phenotype, all subsequent studies only employed females. PRR^RVLM-Null^ exhibited a significant increase in pulse pressure upon DOCA-salt (Figure S3A). Therefore, we sought to measure PWV as an indication of arterial stiffness. As expected, 13 d of DOCA-salt treatment induced a significant increase in pulse wave velocity in both groups, but no significant difference was observed between PRR^RVLM-Null^ and WT (Figure S3B).

**Figure 5.**
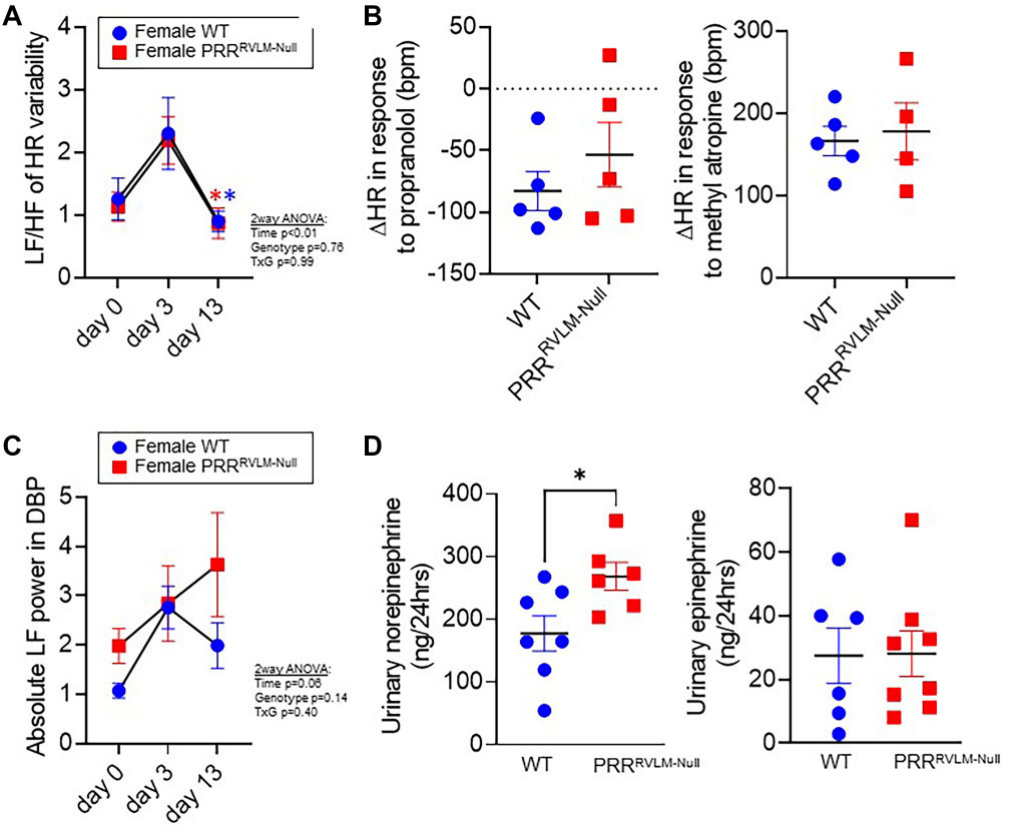
(A) Power spectral analysis of heart rate (HR) variability in female PRR-flox mice subjected to RVLM-targeted microinjection of AAV-CRE GFP (PRR^RVLM-Null^) or control virus AAV-GFP (WT) at 0, 3, and 13 d with deoxycorticosterone acetate (DOCA)-salt hypertension. Data are expressed as mean ± SEM and were analyzed using repeated measures 2-way ANOVA followed by Sidak’s multiple comparison *post-hoc* test. * *P* < .05 versus day 0. (B) Delta heart rate after intraperitoneal injection of propranolol (left; 2 mg/kg) or methylatropine (right; 5 mg/kg). (C) Absolute low frequency (LF) power of diastolic blood pressure variability was calculated at 0, 3, and 13 d with DOCA-salt-hypertension. (D) Twenty-four-hour urine subjected to norepinephrine and epinephrine evaluation at 13 d on DOCA-salt treatment. Data were analyzed using a 2-tailed *t*-test.

To evaluate autonomic balance to the heart, power spectral analyses of HR variability were performed at baseline (day 0), the early stage (day 3), and the late stage (day 13) on DOCA-salt in female WT or PRR^RVLM-Null^. Both groups exhibited an elevated low frequency/high frequency ratio (which estimates a ratio between the sympathetic to parasympathetic activity) in the early stage and a low ratio in the late stage. However, there were no significant differences among the groups (Figure 5A). Supporting these observations, PRR^RVLM-Null^ did not exhibit any significant difference in response to either β-adrenergic or muscarinic blockade with propranolol or atropine, respectively (Figure 5B). PRR^RVLM-Null^ exhibited a diastolic BP variability (a surrogate of sympathetic outflow to resistance arterioles) of 3.67 ± 1.06 compared to 1.99 ± 0.46 in the WT. Unexpectedly, this difference did not reach statistical significance (Figure 5C). Given the variability in these analyses, we sought to measure norepinephrine and epinephrine in urine samples collected at day 13–14. Consistent with the previous findings, female PRR^RVLM-Null^ exhibited significantly elevated urinary norepinephrine (Figure 5D). At day 21, mice were sacrificed, and tissues were weighed and collected for further analysis. No differences in the relative kidney, heart, and spleen weight were found among the groups (Figure S4).

**Figure 6.**
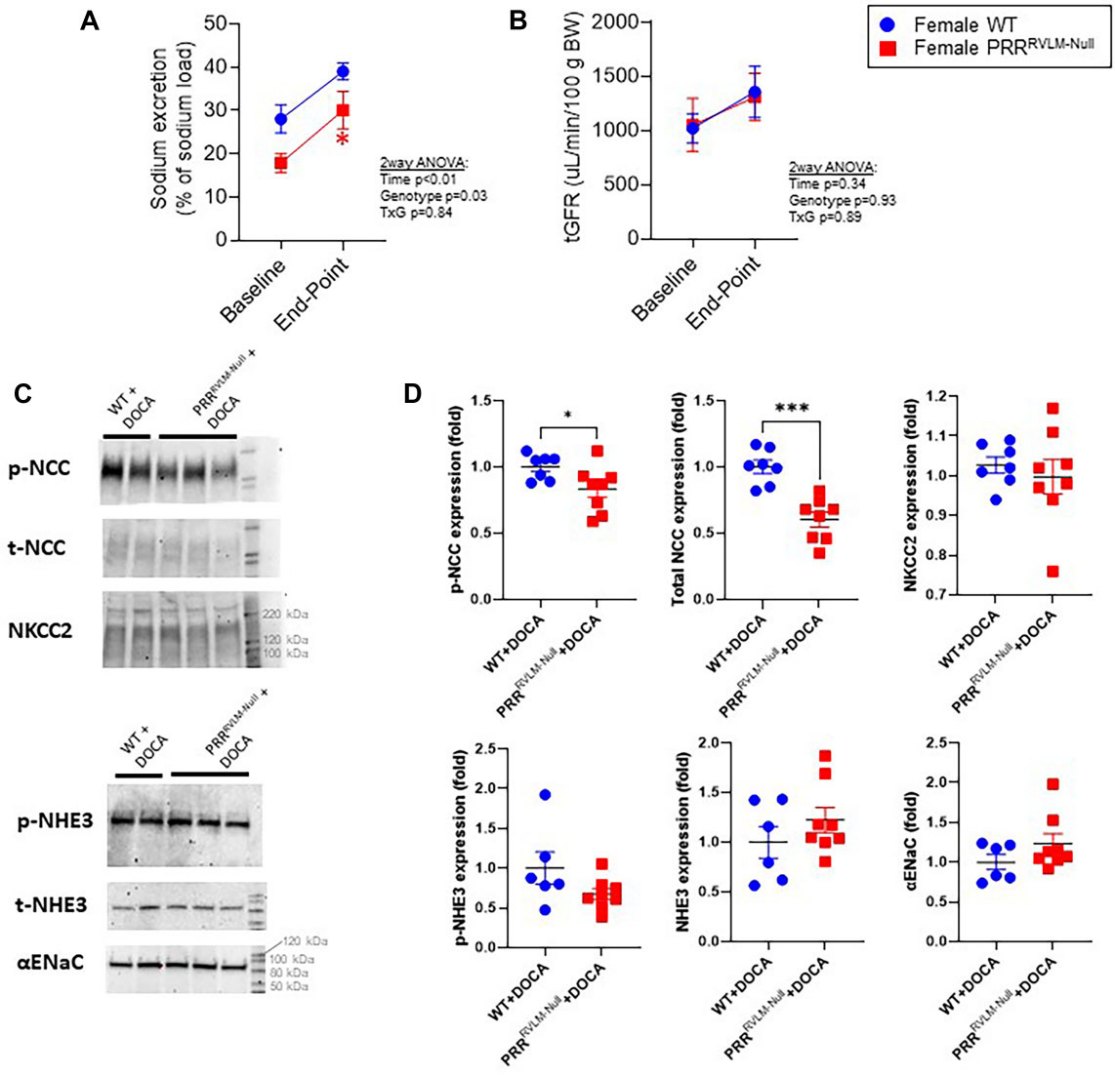
Sodium handling in WT and PRR^RVLM-Null^ females. (A) Urinary sodium excretion 4 h post-intraperitoneal injection of isotonic sodium chloride at baseline and at late-stage on DOCA-salt. (B) Transcutaneous glomerular filtration rate (tGFR) measurements at baseline and late-stage DOCA-salt. (C) Representative western blots. Kidney lysates from DOCA-treated mice were subjected to western blot analysis of p-NCC, total NCC, NKCC2, p-NHE3, total NHE3, and αENAC. A representative blot is shown. Complete blots and loading controls are provided in supplemental Figure S5. (D) Quantification of bands was performed using Image J. Data are expressed as mean ± SEM. Data were analyzed using an unpaired *t*-test.* *P* < .05 and *** *P* < 0.0005 versus WT+DOCA.

We next evaluated whether PRR^RVLM-Null^ exhibited an impaired capacity to excrete sodium. We gave an intraperitoneal injection of isotonic saline (0.9% NaCl) equivalent to 10% of body mass in a separate cohort of mice that were not instrumented with radiotelemeters. Interestingly, PRR^RVLM-Null^ showed an impaired capacity to excrete the excess sodium at baseline and in the late stage of DOCA-salt (Figure 6A). Thus, we hypothesized that PRR^RVLM-Null^ might exhibit either impaired renal function or a sodium-depleted state. To evaluate renal function, we measured GFR by transcutaneous fluorescein isothiocyanate-sinistrin elimination method. No difference in tGFR was observed between groups either at baseline or after DOCA-salt (Figure 6B). Then, we evaluated whether expression of transporters affecting renal sodium reabsorption was altered. Total and phosphorylated expression of various tubular sodium transporters were measured by western blotting. Contrary to our hypothesis, we observed that total and phosphorylated NCC were paradoxically downregulated in PRR^RVLM-Null^. No difference in NKCC2, total and phosphorylated NHE3, and αENaC were observed (Figure 6C and D, Figure S4).

### Genetic Ablation of PRR Targeting the RVLM Augments the Effect of DOCA-Salt on Hydromineral Intake and Output and Mobilizes Fluid Between Intracellular and Extracellular Compartments

**Figure 7.**
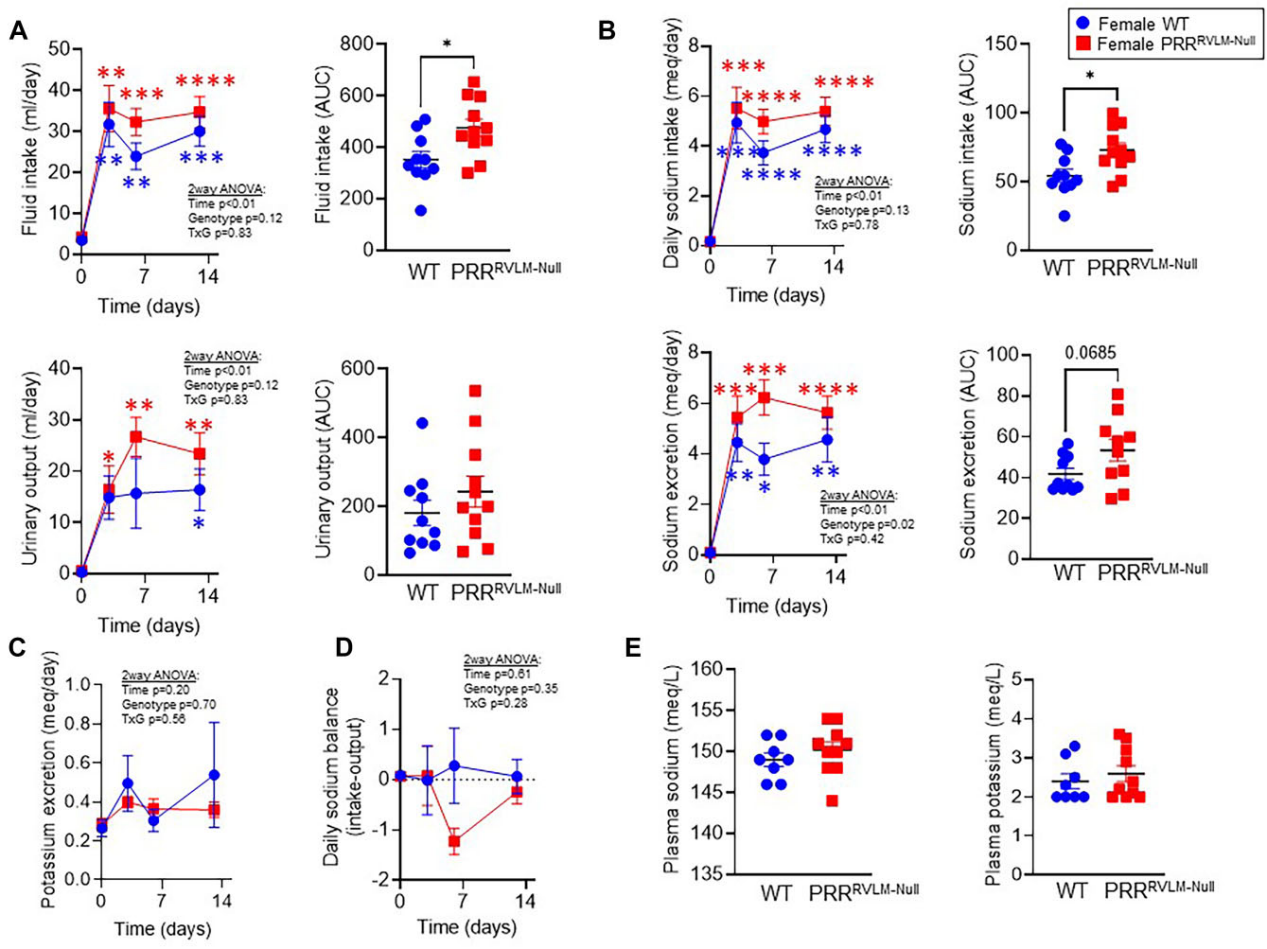
Sodium balance in WT and PRR^RVLM-Null^ females. Mice were placed in metabolic cages for 24-h sodium and volume intake, excretion, and balance evaluation at baseline and at three different stages of DOCA-salt (A–D). (A) Daily volume intake and urinary volume output. (B) Daily sodium intake and urinary sodium output. (C) Daily urinary potassium output. (D) Daily sodium balance calculated as intake minus output. (E) Plasma sodium and potassium levels after DOCA-salt. Data are expressed as mean ± SEM and were analyzed by repeated measures 2-way ANOVA followed by Sidak’s multiple comparison *post-hoc* test. * *P* < .05, ** *P* < .01, and *** *P* < .001 versus baseline. The area under the curve (AUC) was calculated using the trapezoid method and analyzed by an unpaired *t*-test. * *P* < .05 versus WT.

**Figure 8.**
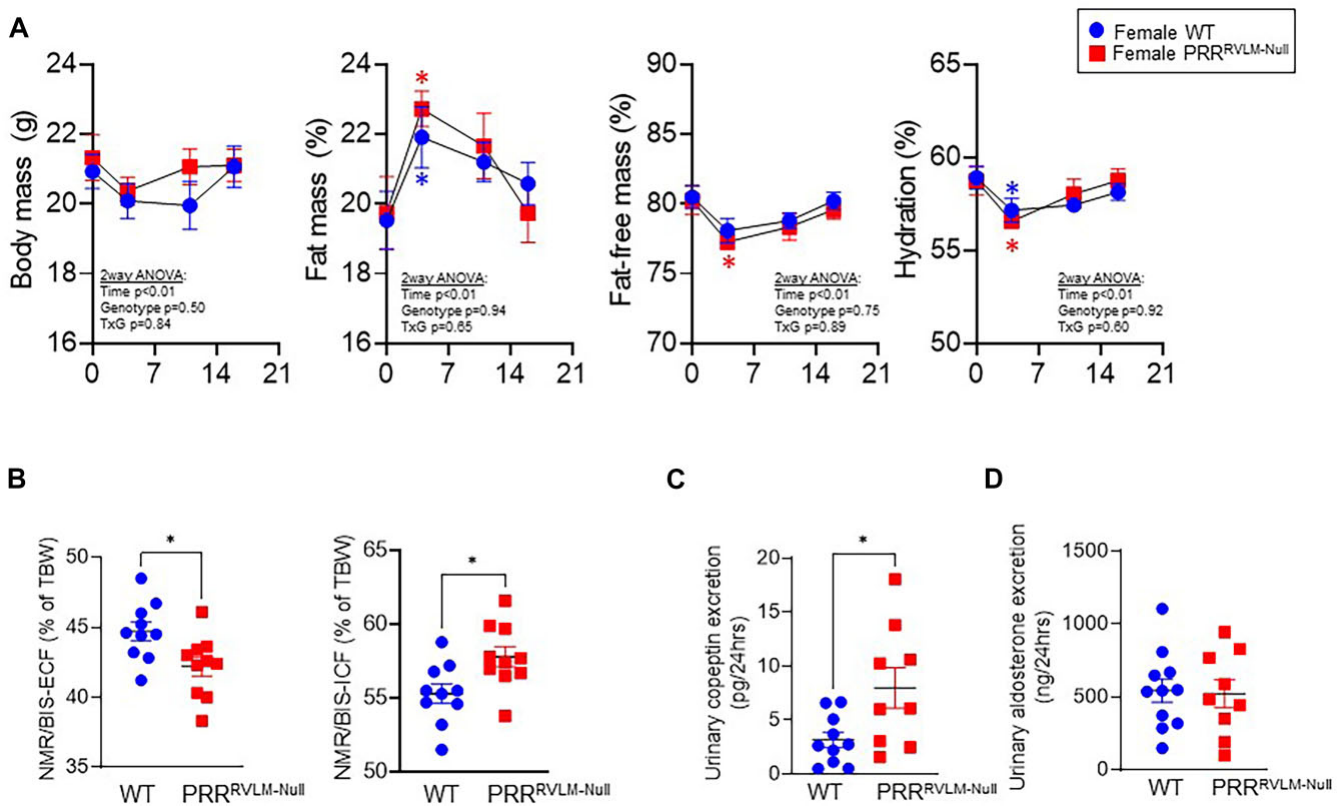
Body composition and fluid compartmentalization in WT and PRR^RVLM-Null^ females. (A) Body composition at baseline and during three stages of DOCA-salt. Body mass, fat mass relative to body mass, fat-free mass relative to body mass, and hydration are shown. Data are expressed as mean ± SEM and were analyzed using repeated measures 2-way ANOVA followed by Sidak’s multiple comparison *post-hoc* test. *P < .05. (B) Extracellular and intracellular fluid relative to total body water. (C and D) Twenty-four-hour urine collection at 13 d with DOCA-salt was subjected to copeptin and aldosterone evaluation. Fluid compartmentalization and urine metabolites were analyzed using an unpaired *t*-test. * *P* < .05 versus WT.

Based on the observations that female PRR^RVLM-Null^ exhibited normal tGFR and downregulated phosphorylated and total NCC, we concluded that lower sodium excretion in response to acute saline challenge might not be due to impaired renal function. We hypothesized that lower sodium excretion after acute load occurs as a response to a sodium and water deficient state, where physiological systems attempt to retain sodium and water to reach hydromineral balance. Therefore, a new cohort was employed to assess fluid and sodium balance at different stages of DOCA-salt. Mice were placed in metabolic cages for a comprehensive 24-h evaluation of sodium input versus output. At baseline, fluid and sodium intake, urinary volume, and sodium excretion between WT and PRR^RVLM-Null^ did not differ. Administration of DOCA-salt induced a prominent increase in sodium and fluid intake as well as urine volume and sodium excretion in both groups. However, female PRR^RVLM-Null^ exhibited an exaggerated increase in fluid and sodium intake and urinary volume and sodium excretion to DOCA-salt (Figure 7A and B). No major differences in potassium excretion, sodium balance, plasma sodium, and potassium concentration were observed (Figure 7C, D, and E). Then, mice were subjected to combined time-domain NMR and BIS to assess body mass and composition, total body water, and fluid distribution between extracellular and intracellular spaces.^34^ No differences between groups in body mass, fat mass, fat-free mass, food and caloric intake, and feces weight were observed at baseline. DOCA-salt administration in both groups induced a transient increase in fat mass concomitant with a decrease in fat-free mass and total body water, which normalized after day 13 (Figure 8A). No difference in food and caloric intake and feces weight was observed (Figure S6). Although no difference in body water was observed among groups, the NMR-BIS analysis revealed that ablation of PRR in the RVLM results in an imbalance between extracellular and intracellular fluids. That is, PRR^RVLM-Null^ exhibited a lower extracellular and higher intracellular fluid (Figure 8B) when compared to WT. We hypothesized that an imbalance between extracellular and intracellular fluid might be sufficient to trigger vasopressin release. Thus, urinary copeptin, a metabolically stable surrogate marker of vasopressin release was quantified. Supporting our hypothesis, 24 h-copeptin levels were significantly elevated in PRR^RVLM-Null^ compared to WT suggesting an elevated vasopressin release (Figure 8C). There was no difference in urinary aldosterone (Figure 8D).

### Transcriptomic Analyses Reveal Canonical Pathways and Putative Gene Candidates Leading to Exaggerated Responses to DOCA-Salt in PRR^RVLM-Null^

**Figure 9.**
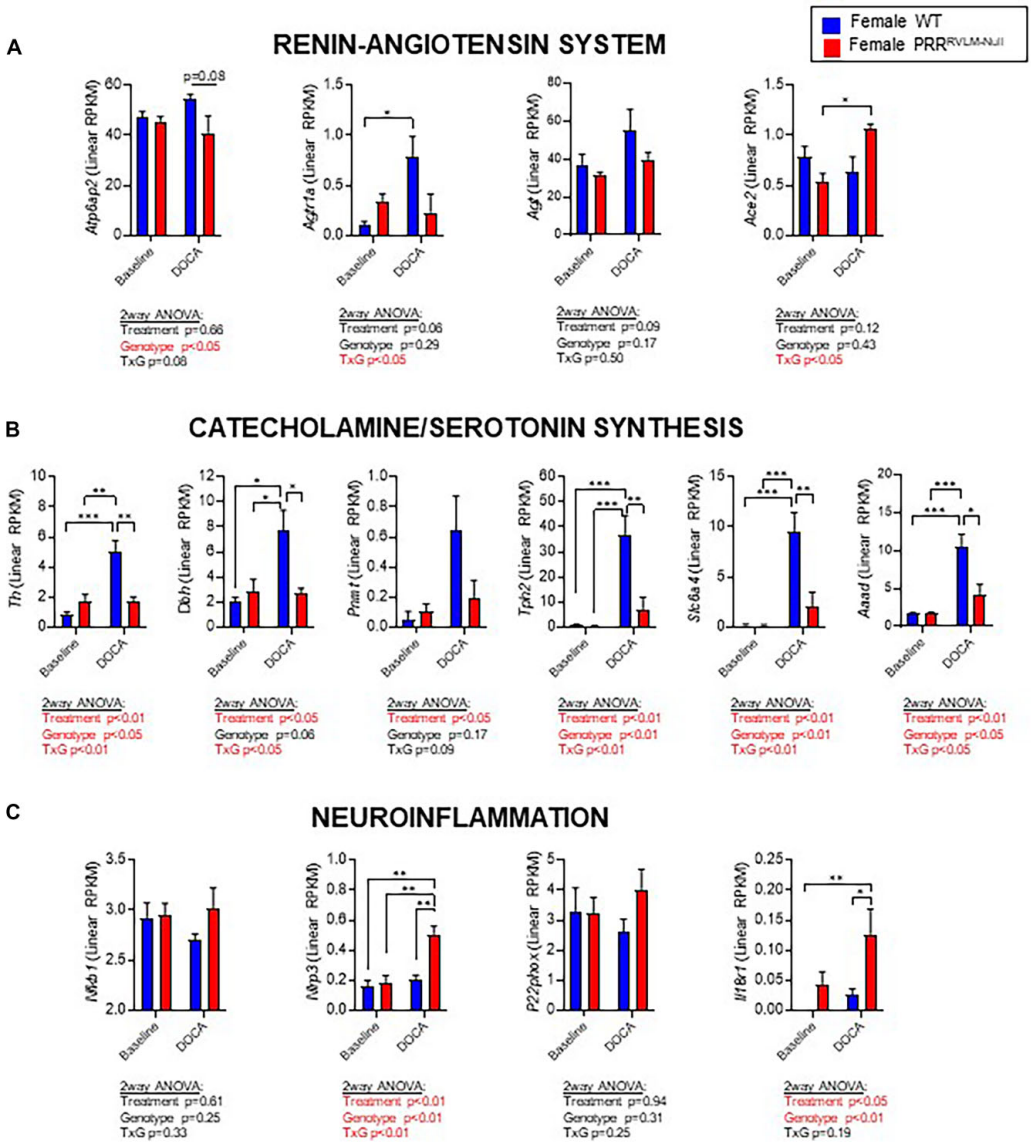
Bulk RNA sequencing data analysis of RVLM brain punches collected from WT and PRR^RVLM-Null^ females at baseline or day 13 with DOCA-salt hypertension. Expression profiles of key genes involved in (A) RAS activation, (B) catecholamine and serotonin biosynthesis, and (C) neuroinflammation are shown. Data are expressed as mean ± SEM and were analyzed using 2-way ANOVA followed by Sidak’s multiple comparison *post-hoc* test. * *P* < .05, ** *P* < .01 and *** *P* < .001.

Using the Palkovits brain punch technique, we obtained total RNA from female RVLM at baseline and after 13 d of DOCA-salt. Samples were subjected to bulk RNA sequencing to identify putative candidates that could explain physiological outcomes. Differential expression analyses using the generalized-linear regression approach revealed an upregulation of 434 genes and downregulation of 128 genes in DOCA-salt HTN compared to the baseline control. Ablation of PRR in DOCA-salt induced upregulation of 258 genes and downregulation of 147 genes compared to mice targeted with a control virus (Figure S7A–C). RNA-seq gene expression data were then analyzed using Ingenuity Pathway Analysis to infer the influence of PRR deletion on biological networks and signaling pathways. The top 5 canonical pathways and upstream regulators are listed in Figure S7D and E. Then, we sought to selectively evaluate key candidate genes individually. First, as expected, there was a trend toward a decrease (*P* = .08) in PRR expression in DOCA-salt-treated PRR^RVLM-Null^ compared to WT (Figure 9A). Interestingly, there was no difference at baseline, presumably due to insufficient time to induce a complete extinction of mRNA from every cell in the sample that express PRR. In WT mice, DOCA-salt increased expression of several RAS genes, including *Agtr1a*, catecholamine synthesis (*Th, Dbh, Pnmt*) and serotonin biosynthesis (*Tph2, Slc6a4, Aaad*) (Figure 9A and B). PRR ablation prevented the effects of DOCA-salt on several of these genes. Interestingly, PRR^RVLM-Null^ exhibited upregulation of *Ace2*, considered a protective arm of the RAS. Moreover, the lack of PRR in the RVLM resulted in elevated genes involved in inflammatory responses, including *Nfkb1, Nlrp3, P22phox*, and *Il18r1* (Figure 9C). These data might indicate that ablation of PRR in the RVLM is sufficient to suppress local RAS activation and induction of catecholamine and serotonin biosynthesis in the RVLM by DOCA-salt. However, the activation of neuroinflammatory signals might trigger the exacerbated fluid and pressor responses to DOCA-salt in PRR^RVLM-Null^.

## Discussion

The main finding of this study is that the localized ablation of PRR in the RVLM region results in a sex-dependent phenotype characterized by exacerbated dipsogenic, natriuretic, and pressor responses to DOCA-salt during the late “maintenance” phase of the model. In addition, this phenotype was also associated with exacerbated dipsogenic response, decreased relative distribution between extracellular and intracellular fluid compartmentalization, elevated urinary copeptin (an index of arginine vasopressin), and increased sympathetic tone to the kidney and resistance arterioles.

The physiological outcomes of brain RAS activation are highly dependent on the neural circuits involved. This study was focused on the RVLM, a key region of cardiovascular regulation and sympathetic control, where we previously identified renin promoter activity.^25^ The RVLM region is situated within the blood-brain barrier; although it is unlikely that circulating ANG-II can reach the RVLM neurons directly—especially in the context of DOCA-salt HTN where circulating levels of ANG-II are low—the RVLM remains subject to trans-endothelial signaling and thus potential ANG-II transcytosis.^7,35,36^ The PRR has been proposed as a key player in the generation of ANG-II in several tissues by facilitating the non-proteolytic activation of (pro)renin.^37^ Studies designed to investigate the specific role of PRR in specific brain regions such as the subfornical organ or paraventricular nucleus emerged recently.^18,38,39^ Using in situ hybridization technique, we observed that PRR is widely expressed in RVLM and is present in a wide variety of cell types, including glutaminergic and GABAergic neurons as well as in microglia. Stereotactic injections of recombinant mouse prorenin targeting the RVLM resulted in acute pressor responses, indicating that the RVLM region contains the machinery to activate and respond to exogenous prorenin. Moreover, recombinant prorenin elicited intracellular signaling and generation of reactive oxygen species in brainstem neurons in vitro. Whether these effects of prorenin are RAS-dependent still needs to be evaluated. Then, we stereotactically delivered AAV-CRE into the RVLM of mice carrying floxed PRR alleles to perform region-specific gene deletions. Since previous studies reported that the functions of PRR are sex-dependent, we included both sexes in this study.^40–42^ Likewise, sex differences in the development of DOCA-salt HTN include dimorphism in BP, vasopressin release, vascular reactivity, inflammatory profile, baroreflex function, and importantly the brain RAS activity. ^43–48^ Similarly, evidence supports that development of DOCA-salt HTN is influenced by the estrous cycle.^49^ We acknowledge this represents a potential confounding factor that was not considered in the study. Contrary to our expectations, males lacking PRR did not exhibit any difference in BP compared to the WT control upon DOCA-salt challenge. In contrast, upon DOCA-salt stimulation, female PRR^RVLM-Null^ mice displayed significantly lower diastolic BP during the early phase (first week). Subsequently, female PRR^RVLM-Null^ mice on DOCA-salt displayed an exacerbated increase in systolic BP during the late “maintenance” phase (after the second week). These experiments led us to two main conclusions. First, the unexpected exacerbation of BP increases after the delayed development of DOCA-salt HTN in female PRR^RVLM-Null^ mice suggests that different roles of PRR might exist depending on the cell type and brain region involved. Second, it is well-accepted that the development of HTN to DOCA-salt involves different physiological mechanisms that change throughout time. During the initial days of DOCA-salt treatment, fluid retention due to an imbalance in renal sodium handling concomitant with excessive salt intake is critical for the full establishment of HTN. Then, the slow increase of BP and sustained HTN, which occur during the next few weeks, is thought to be mediated by neural and humoral factors, including elevated sympathetic tone to resistance arterioles and vasopressin release, respectively.^50^ We considered that in the model DOCA-salt HTN, the role of PRR in the RVLM is bi-phasic given that RVLM-specific ablation of PRR in the early developmental stage of DOCA-salt HTN is protective, while during the late maintenance stage, seems to be detrimental. Importantly, the increase in BP in the female PRR^RVLM-Null^ mice might be linked to sympathoexcitation, as the power spectral analysis of diastolic BP variability was elevated in these animals. We acknowledge that power spectral analysis is an indirect measurement of sympathetic drive. Thus, alternatively, we also measured urine norepinephrine, which was elevated in the female PRR^RVLM-Null^ mice. Ideally, renal nerve recording might confer additional direct measurement of sympathetic drive. However, this technique becomes a limitation given the need for anesthetics and the size restraint of mice. An alternative mechanism causing elevated BP can be explained by the observation that female PRR^RVLM-Null^ mice exhibited exacerbated salt and water intake in response to DOCA-salt. There was no significant change in the daily sodium balance, presumably because there was a compensatory reduction in the expression and activity of sodium transporters, leading to elevated urinary excretion. It has been reported that conditions such as DOCA-salt HTN induces a shift of fluid from the extracellular to intracellular spaces.^51^ Similarly, our study revealed that females lacking PRR showed a reduction of fluid in the extracellular and increase in the intracellular compartment. Cianci et al. have reported that the increase in total body water content in hypertensive patients is attributable to the expansion of the intracellular fluid. Moreover, intracellular water correlates with BP.^52^ It has been also indicated that hypertensive patients also exhibit a reduction in the extracellular fluid volume.^53^ Given that extracellular dehydration stimulates drinking and vasopressin release, it might be possible that the altered fluid distribution explains in part the elevated urinary copeptin levels and exacerbated drinking response to DOCA-salt in PRR^RVLM-Null^ mice.^54^

To identify key genes affected by the removal of PRR in the RVLM, we employed transcriptomic approaches in brain punches collected from the RVLM of female WT or PRR^RVLM-Null^ mice at baseline and during the maintenance late phase of DOCA-salt. Using Ingenuity pathway analysis, which allows a comprehensive gene expression data analysis to infer the underlying causes of the observed changes, we identified three important canonical pathways that could be implicated in this phenomenon: (1) G-protein coupled receptor signaling, (2) DNA methylation and transcriptional repression, and (3) phagosome formation. PRR appears to be essential for the maintenance of critical cellular functions as it is required for the assembly of the lysosomal proton-transporting V-type ATPase, protein degradation, and phagolysosome formation.^55^ Interestingly, we identified POU4F1 as the main upstream regulator. It has been shown that POU4F1 plays a key role in the development of the nucleus ambiguus, and mice lacking POU4F1 die prematurely due to defects in this nucleus.^56^ Thus, the absence of PRR might be lethal for certain cell types within the RVLM. Future studies that use advanced techniques to ablate specific cell types and neuronal populations within the RVLM region, such as the nucleus ambiguus, would be required. Differential analysis of genes that are associated with the regulation of the RAS and sympathetic activity in the RVLM as well as inflammatory cascades were performed. Although female PRR^RVLM-Null^ mice exhibited higher BP when the RVLM punches were collected, we observed a paradoxical decrease in the expression of “detrimental” RAS genes and elevated expression of “protective” RAS genes. This implies that despite the ablation of PRR resulting in the local inhibition of the RAS in the RVLM, this protective effect was insufficient to counterbalance the hypertensive responses elicited by alternative mechanisms. Moreover, the ablation of PRR upregulated genes associated with inflammation. Therefore, we hypothesize that ablation of PPR might target POU4F1 and impair mechanisms involving G-protein signaling, DNA methylation, and phagosome formation leading to a localized inflammatory response in the brainstem. We acknowledge that there are certain limitations. First, the RVLM is not a defined anatomical structure with clear boundaries and is defined as a region within the medullary reticular formation. This region comprises several subregions and heterogeneous cell types, including the C1 adrenergic cell group, cholinergic neurons, and many others.^26^ Importantly, PRR is expressed in almost every cell type, including the microglia within the RVLM.^24^ Studies have shown the presence of PRR in other cell types in the brain. For instance, PRR is expressed in astrocytes and plays a role in oligodendrogenesis and endothelial cell polarity. Thus, we consider a possible role for PRR to not only be expressed in neurons and microglia but also in astrocytes, oligodendrocytes, and endothelial cells. Although this was not directly measured, analysis of genes related to the functions of these cells by RNA sequencing did not suggest significant alterations.^21,57^ Given the lack of clear anatomical boundaries, the histological confirmation of the correct delivery of the viruses has been challenging. Therefore, transient pressor responses to small amounts of glutamate, an excitatory neurotransmitter, were used as criteria to confirm the correct injection site. In addition to the AAV-GFP control group, we considered other controls such as off-target AAV-cre injections into areas in the brain presumed to play no role in BP or AAV-cre injections in wildtype mice. However, we felt that these additional experiments would not add significant impact to the study as the results would largely be negative. We consider it likely that complete ablation of PRR was not achieved. Our previous experience in other brain regions indicates that the efficiency of gene recombination never reaches 100%. In this study, the reduction in PRR expression in the RVLM punches was ∼30%, but it did not reach statistical significance (*P* = .08). Of note, however, the area of the brain punch is relatively bigger than the RVLM; thus, some surrounding tissue that did not undergo viral transduction and thus cannot undergo PRR ablation was sampled. Thus, the decrease in PRR expression is likely an underestimate, perhaps largely so. Additionally, we recognize that the serotype of the AAV used might not possess the most efficient tropism to infect the variety of cells comprised in the RVLM. Finally, we could not conduct cell-specific ablation of PRR in the RVLM region. Given the ubiquitous expression of PRR and the heterogeneity of the RVLM region, it is likely that cell-specific ablation of PRR could lead to different physiological outcomes. Future studies will address whether conditional ablation of PRR in cholinergic neurons, catecholaminergic C1 neurons, non-C1 neurons, or glial cells recapitulates the phenotype of PRR^RVLM-Null^ mice.

In conclusion, we demonstrated that PRR in the RVLM region plays an important role in BP and hydromineral balance in a model of low plasma renin HTN. Importantly, the role of PRR is sex-dependent and cell-type-specific. Given the extensive and ubiquitous expression of PRR in brain regions of cardiovascular regulation, it is of utmost importance to consider that there might be off-target effects in therapeutic strategies targeting PRR.

## Supplementary Material

zqad043_Supplemental_Files

## Data Availability

The bulk RNA sequencing data have been deposited to the NCBI Gene Expression Omnibus accession series with the identifier 235234. The data that support the findings of this study are available from the corresponding authors upon reasonable request.
