# Supplementary material for: Genetic Ablation of Prorenin Receptor in the Rostral Ventrolateral Medulla Influences Blood Pressure and Hydromineral Balance in Deoxycorticosterone-Salt Hypertension
Source: Function (Oxf). 2023 Aug 7;4(5):zqad043. doi: 10.1093/function/zqad043 (PMC10440998; doi:10.1093/function/zqad043)
Supplement: zqad043_Supplemental_Files [file zqad043_supplemental_files.zip › PPR-Manuscript Supplemental-v4-FINAL.docx]

**Genetic Ablation of Prorenin Receptor in the Rostral Ventrolateral Medulla Influences Blood Pressure and Hydromineral Balance in Deoxycorticosterone-Salt Hypertension**

Natalia M. Mathieu^1*^, Eva M. Fekete^1*^, Patricia C. Muskus^1*^, Daniel T. Brozoski^1^, Ko-Ting Lu^1^, Kelsey K. Wackman^1^, Javier Gomez^1^, Shi Fang^1^, John J. Reho^1,2^, Connie C. Grobe^3^, Ibrahim Vazirabad^1^, Gary C. Mouradian, Jr.^1,4,6^, Matthew R. Hodges^1,6^, Jeffrey L. Segar^3^, Justin L. Grobe^1,2,4,5,6^, Curt D. Sigmund^1,4,6^, Pablo Nakagawa^1,4,6^

^1^Department of Physiology, Cardiovascular Center, Medical College of Wisconsin, Milwaukee, WI, United States

^2^Comprehensive Rodent Metabolic Phenotyping Core, Medical College of Wisconsin, Milwaukee, WI, United States

^3^Department of Pediatrics, Medical College of Wisconsin, Milwaukee, WI, United States

^4^Cardiovascular Center, Medical College of Wisconsin, Milwaukee, WI, United States

^5^Department of Biomedical Engineering, Medical College of Wisconsin, Milwaukee, WI, United States

^6^Neuroscience Research Center, Medical College of Wisconsin, Milwaukee, WI, United States

*Equal contribution

Running title: PRR in the RVLM

Corresponding Authors:

Pablo Nakagawa

Department of Physiology

Medical College of Wisconsin

8701 Watertown Plank Rd

Milwaukee, WI 53226

United States

[pnakagawa@mcw.edu](mailto:pnakagawa@mcw.edu)

-or-

Curt D. Sigmund

Department of Physiology

Medical College of Wisconsin

8701 Watertown Plank Rd

Milwaukee, WI 53226

United States

[csigmund@mcw.edu](mailto:csigmund@mcw.edu)

**Methods**

Acute studies: Two acute studies were conducted in male C57BL/6J mice. Experiment 1: To study whether pharmacological blockade of prorenin receptor (PRR) in the brain exerts anti-hypertensive effects in mice with established hypertension (HTN), an acute intracerebroventricular (ICV) infusion of prorenin receptor antagonist, PRO20, was performed in mice with established deoxycorticosterone acetate (DOCA)-salt HTN. Briefly, 12-week-old male C57BL/6J mice were instrumented with a radiotelemetry catheter to monitor systolic blood pressure (BP) and a stainless-steel cannula into the lateral ventricle for the acute ICV injections of either control scramble peptide (3 nmol), PRO20 (3 nmol), or losartan (18 nmol, cat#SML3317, Sigma Aldrich) dissolved in artificial cerebrospinal fluid (aCSF), respectively. The stereotactic coordinates for the ICV canulation were: 1.0 mm mediolateral, 0.3 mm caudal to bregma, and 2.5 mm ventral from the dorsal surface of the skull as validated previously.^1^ Animals were allowed to recover for 10 days from surgeries. Systolic BP was measured at baseline and post-50 mg of DOCA (Sigma Aldrich, Saint Louis, MO) pellet implantation. Mice received 0.15 M NaCl saline as the only choice of water thereafter. Awake and freely moving mice were subjected to acute ICV administration of either scramble peptide, PRO20, or losartan. Two microliters of drugs were infused at a rate of 0.2 μL/min for 5 minutes using a peristaltic pump. BP was recorded for 60 minutes following infusion. The area under the curve of BP tracing was calculated using the trapezoidal method. Experiment 2: To study whether administration of recombinant prorenin targeting the rostral ventrolateral medulla (RVLM) region exerts pressor responses acute unilateral microinjection of aCSF, recombinant prorenin (0.3 nmols), or angiotensin II (ANG II, 0.3 nmols) targeting the RVLM were performed, as aforementioned. Once the BP was stable, 1-minute baseline recording was collected and subsequently 30 nL of either aCSF, recombinant prorenin, or ANG II were delivered, and BP was recorded during the subsequent 5 minutes. Changes in systolic BP in response to drug injection were calculated and the area under the curve was calculated using the trapezoidal method. After the treatments, 5 nL of fluorescent microspheres were delivered and the brains were sectioned to confirm the correct placement of the injector in the RVLM region histologically.

Neuronal cells culture: Neuronal cells from neonatal brainstem were cultured to evaluate responses to recombinant prorenin. Five to seven brainstems were collected in aseptic conditions from postnatal day 1-3. Under the hood, the meninges and white matter were removed. Tissues were chopped with a sterile blade and incubated in hibernate A medium containing 2 % papain and 5 mM L-cysteine for 30 minutes at 37° C. Then, the tissues were disrupted by pipetting up and down with a fire-polished glass Pasteur pipette. Approximately 4x10^5^ cells were plated in poly-D-lysine coated wells containing Neurobasal A plus medium containing 2 % B-27 Plus supplement, antibiotic/antimycotic, and Glutamax supplement. Half of the media was replaced with new media every third day. Experiments were conducted after day 4. To measure activation of ERK signaling cells were treated with recombinant prorenin (Anaspec cat # As-72174) for 0, 30, and 60 minutes. After incubation, cells were washed with ice-cold PBS and lysed with RIPA buffer (ThermoFisher Scientific, cat#89900) containing protease and phosphatase inhibitors with subsequent sonication. Samples were centrifuged and supernatants were stored for further analysis. Western blotting was performed as we described below. To determine the effect of prorenin on superoxide production in brainstem neurons cells were treated with either vehicle of recombinant prorenin for 6 hours. Cells were homogenized with an ice-cold lysis buffer containing 20 mM KH_2_PO_4_, 1 mM EGTA, and protease inhibitors. Then, cell homogenate was incubated in a pH 7.4 buffer containing 1 mM lucigenin, 50 mM KH_2_PO_4_, 1 mM EGTA, and 150 mM sucrose. Superoxide production in response to 1 mM NADPH was quantified using a luminometer (FLUOstart Omega Microplate reader, BMG Labtech, Cary, NY).

Telemetric recordings: BP, heart rate, and activity were measured in awake animals as previously shown.^2^ Briefly, mice were anesthetized with ketamine (90 mg/kg i.p.)/xylazine (5 mg/kg i.p.) and ^3^a radiotelemetry catheter (Data Science International, Model TA11PA-C10) was inserted in the left carotid artery and advanced into the transverse aorta. The catheter was secured with three sutures and the incision was sutured with 6-0 surgical silk. Analgesics (meloxicam) were given immediately after the surgery and again 24 hours later. After surgery, the animals were single housed. After 10 days of post-surgical recovery BP, heart rate, and activity were recorded for 10 seconds every 5 minutes for 24 hours. Data from each animal were averaged daily, hourly and during the light (5 AM to 7 PM) and dark phase (7 PM to 5 AM).

Power spectral analyses: Continuous high frequency (2,000 Hz) radiotelemetry 24-h recording at baseline, day 3 and 13 on DOCA-salt were obtained. Power spectral analysis for heart rate variability and blood pressure variability was performed as previously described.^3^ Briefly, beat-by-beat heart rate and blood pressure time series were derived from the blood pressure waveforms and converted to an equidistant sampling rate using cubic spline interpolation. The very low frequency (VLF, 0.02-0.20 Hz), low frequency (LF, 0.2-0.6 Hz) and high frequency (HF, 1.0-5.0 Hz) were derived from fast Fourier transform of the equidistant heart rate time series. The relative LF and HF were calculated as the relative value of each power component in proportion to the total power minus the VLF component. The LF/HF ratio of heart rate variability was used as an indication of sympathetic/parasympathetic balance to the heart. The LF power of the diastolic blood pressure was employed as a surrogate parameter of the sympathetic tone to the resistance arterioles.

Biochemical assays: Urine electrolytes were measured using a flame photometer (Jenway PFP7 or BWB XP) and blood electrolytes were determined using an i-STAT portable blood analyzer and CHEM8+ cartridges (Abbott Laboratories, IL).

In situ hybridization: Euthanasia was conducted using pentobarbital overdose. Animals were then transcardially perfused with ice-cold phosphate-buffered saline (PBS) containing 0.1% heparin and then fixed with 4% paraformaldehyde. The brains were cryoprotected with 30% sucrose for 3 days. The brains were placed into a cryomold containing Tissue-Tek optimal cutting temperature compound (O.C.T., Sakura) and snap frozen in dry ice and 4-methylbutane (Sigma Aldrich). The brain was cryosectioned coronally at 10-14 µm and stored at -80°C until use. Endogenous mRNA was detected using the *in situ* hybridization technique RNAscope® Assay (ACDBio, Newark, CA) using the manufacturer protocols. The following probes were used: PRR (Mm-atp6ap2, 429931, ACD Bio), NeuN (Mm-Rbfox3, 313318, ACDBio), Iba-1 (Mm-aif1, 319148, ACD Bio), GABA vesicular transporter (Mm-slc32a1, 319191, ACD Bio), Vglut (Mm-slc17a6, 319171, ACD BioPeptidylprolyl isomerase B (Ppib, Mm-Ppib – 313911, ACD Bio) and 4-hydroxy-tetrahydrodipicolinate reductase (dapB, 310043, ACD Bio) probes were used as positive and negative controls, respectively. RNAscope® assay protocol was followed for all other amplifications and development steps. Slides were counterstained in hematoxylin dye, Gill No. 1 (GHS132, Sigma Aldrich) and cover slipped with EcoMount (EM897L, Biocare Medical). Images were taken using a fluorescent microscope system (Keyence Model BZ-X800, Itasca, IL).

Assessment of aortic stiffness by non-invasive pulse-wave velocity (PWV): PWV was measured at baseline and day 13 post DOCA-salt using a Doppler ultrasound system (Mouse Doppler TM, Indus Instruments, Webster, TX) as previously described.^4^ Briefly, mice were anesthetized with 1.5-2.0 % isoflurane and prepared for non-invasive PWV measures. The thoracic and abdominal aortic regions were shaved using hair-removal cream (Nair, Church & Dwight, Ewing, NY). Then, animals were placed on a temperature/electrocardiogram (ECG) platform in a supine position and paws were taped to the ECG electrodes. One 20 Mhz Doppler probe was placed on the skin above the thoracic aorta region and another probe on the skin above the abdominal aorta. Transit times of pulse pressure waves were calculated and the distance between the abdominal and descending aorta was measured using a ruler. PWV is calculated by dividing the abdominal-to-descending aorta distance and the transit time.

Combined nuclear magnetic resonance (NMR) and bioimpedance spectroscopy (BIS): Body composition and intracellular/extracellular fluid distribution were assessed by a combined time-domain NMR-BIS method as previously shown.^5^ First, body composition (fat and lean tissues, and water content) was measured in a non-invasive way using a rodent-sized time-domain NMR system (Bruker, Model LF110, Billerica, MA). The system was previously calibrated using mixes of oil for fat tissue, lean chicken breast meat for lean tissue, and saline or water for free fluid. Fat-free mass was calculated as the total body mass minus fat mass and total body water was calculated as 73.2 % of fat-free mass. Immediately after, mice were subjected to BIS using an ImpediVET BIS1 system (ImpediMed, Carlsbad, CA). Briefly, animals were anesthetized with 1.5-2.0 % isoflurane, and electrodes were placed on the back of the head (located at an imaginary intersection from a line connecting the front of the ears with the midline of the animal), and on the lower back (located at another imaginary intersection where the muscle of the thighs of the animal meet the body midline). Additional electrodes are then placed 1-2 cm anterior to the head electrode and 1-2 cm posterior to the lower back electrode. The following settings were used to assess fluid compartmentalization: density = 1.05; proportion = 1; hydration = 0.732; males: RhoE = 998.9, RhoI = 1220.2/females: RhoE = 586.9, RhoI = 756.8. Linear correlations among fluid compartments and theoretical “lengths” between emitting and recording electrodes were employed to obtain corrected BIS-derived total body water, extracellular fluid, and intracellular fluid values.

Acute saline challenge experiments: Volume and saline challenge studies were conducted at baseline and day 21 as previously described.^6^ The animal received an intra-peritoneal injection of 0.9 % NaCl saline that is 10% of their body mass as volume/sodium challenge and immediately placed in a metabolic cage (Hatteras Instruments, MMC100, Cary, NC). Four-hour urine volume and sodium concentration were measured using a flame photometer (Jenway PFP7 or BWB XP). All urine samples were processed by a blinded operator.

Assessment of renal function: Transcutaneous glomerular filtration rate (tGFR) was measured in conscious mice using the transcutaneous measurement of the elimination kinetics of fluorescein isothiocyanate (FITC)-sinistrin as described by Schreiber et al.^7^ Briefly, animals were anesthetized with 1.5-2.0 % isoflurane to inject FITC-sinistrin retroorbitally. Then, an area of 2 cm x 2 cm was cleaned of hair by a hair clipper and Nair on the dorsal skin, and a transdermal fluorescent monitor (MediBeacon, Creve Coeur, M) was closely attached to the skin for continuous data collection. The half-life (t_1/2_) of FITC-sinistrin was determined in the following 120 minutes. The calculation of tGFR was performed by a blinded operator using the following equation:

$$GFR \left[ \mu L\cdot min\cdot100g body mass \right]=\frac{14616.8 [\frac{\mu L}{100g body weight}}{t_{1/2} \left( FITC-sinistrin \right)[min]}$$

Western blotting: Western blotting was performed to detect 1) PRR in brain punches, whole hypothalamus, brainstem, and cortex, 2) phosphorylated and total ERK, and 3) renal sodium transporters in the kidney homogenate. To detect PRR, 20 mg of frozen tissue were homogenized in radioimmunoprecipitation assay buffer (RIPA buffer) lysis buffer (Santa Cruz Biotechnology, Dallas, TX) containing proteinase and phosphatase inhibitors (Roche, Basel, Switzerland) on ice. Protein concentration was determined using the BCA method (Pierce Thermo Scientific, Rockford, IL). Thirty μg of protein were prepared in 4x Laemmli Sample Buffer (Bio-Rad Laboratories, Hercules, CA) and boiled for 10 minutes. Proteins were separated in a 4-12% gradient gel and then transferred to a polyvinylidene fluoride or polyvinylidene difluoride (PVDF) membrane. Membranes were stained with Ponceau red to confirm equal protein loading. Membranes were blocked with phosphate buffer containing 5% bovine serum albumin (BSA) and 0.1% polysorbate 20 (Tween-20) for 1 hour at room temperature. The membranes were incubated overnight at 4°C with anti-atp6ap2 (1:500, Sigma Aldrich, Cat #HPA003156) or anti-tdTomato (1:1,000, Abcam Cat #ab213511). Membranes were washed five times (five minutes each) and exposed to a horseradish peroxidase (HRP)-conjugated secondary antibody (Santa Cruz Biotechnology, Dallas, TX) for 1 hour at room temperature. Membranes were developed using ECL Western Blotting Substrate (ThermoFisher Scientific, Waltham, MA). Chemiluminescence was detected using a ChemiDoc Imaging Systems (BioRad Laboratories, Hercules, CA) and band densities were quantified using Image J software (version 1.51). Membranes were stripped with Restore™ Western Blot Stripping Buffer (ThermoFisher Scientific, Waltham, MA). All analyses and data acquisition were performed by a blinded operator. To detect phosphorylated and total ERK, a similar protocol was employed except the primary antibodies were anti-phospho-p44/42 (Thr202/Tyr204)(1:1,000, Cell Signaling Cat #4370) or anti-total ERK (1:1,000, Cell Signaling Cat #4695). A protocol described by Veiras et al.^8^ was used to detect sodium transporters in the renal cortex. Briefly, kidney cortex was homogenized in ice-cold buffers containing 5% sorbitol buffer, 0.5 mM disodium EDTA, and 5 mM histidine-imidazole buffer (pH=7.5), 0.2 mM phenylmethylsulfonyl fluoride, 9 μg/mL aprotinin, and 5 μl/mL of a phosphatase inhibitor cocktail (Sigma-Aldrich, P0044)]. Then, the homogenates were centrifuged at 2,000 x g for 10 min at 4° C and the supernatant was stored at -80°C. Then, the immunoblotting protocol described above with the following antibodies was performed: phosphorylated NCC (1:1,000; PhosphoSolutions Cat #p1311-53), total NCC (1: 200; StressMarq Cat #SPC-402), NKCC2 (1:1,000; StressMarq Cat #SPC-401), phosphorylated NHE3 (1:1,000; Santa Cruz Biotechnology Cat #sc-53962), total NHE3 (1:1,000; Santa Cruz), and alpha ENaC (1:2,500; StressMarq Cat#SPC-403). Membranes were developed and analyzed using the aforementioned method.

Bulk RNA sequencing: WT and PRR^RVLM-Null^ females were euthanized at 13 days on DOCA-salt HTN. Brains were immediately harvested and frozen in 2-methyl butane on dry ice and stored in -80° C. A separate cohort of animals without DOCA-salt treatment were euthanized after 3 weeks post RVLM-targeted microinjections of either AAV GFP or AAV CRE GFP. Palkovits technique was used to obtain RVLM tissue samples as previously described.^9^ Briefly, the brains were embedded in Tissue-Tek O.C.T compound (Sakura) in a cryostat and 250 μm sections were collected between AP: -7.2 to -6.5 mm coordinates caudal from bregma. Bilateral brain punches of the RVLM region were collected using a 1-mm needle (Stoelting) and maintained frozen until processing. Total RNA was extracted using TRIzol (Thermo Fisher Scientific). Total RNA was quantified using spectrophotometry (NanoDrop, Thermo Fisher Scientific) and confirmed using fluorometric methods (Qubit, Invitrogen). Samples were then submitted to Genomic Sciences & Precision Medicine Center at MCW for library preparation and RNA sequencing analysis. A total of 26 samples were submitted (WT baseline=6, PRR^RVLM-Null^ baseline=8, WT+DOCA-salt=6, PRR^RVLM-Null^+DOCA-salt=6). Samples were subjected to quality control using a fragment analyzer. Library preparation was conducted using Low input SMART-seq stranded kit (Takara). Libraries were quantified using Kapa quantification kit with MiSeq 50 cycle run (Kapa Biosystems). A total of 15 samples passed all quality controls: WT (baseline: 4, DOCA-salt: 3) and PRR^RVLM-Null^ (baseline: 4, DOCA-salt: 4). Paired-end sequencing was conducted with NovaSeq at 2 x 100 bp reads targeting 40-50 million reads per sample. Alignment to the mouse reference (Gencode vM23) was performed using the STAR aligner and MAPR-Seq workflow version 3.0. Quality control were performed using FastQC and RSeQC packages. Differential expression analyses were performed using the following threshold limits: CPM>1 in at least 1 samples, FDR (adjusted p-value) <=0.05, FC=2, and protein coding gene biotype. EdgeR was used to analyze DE between groups using the GLM approach. Data were visualized with volcano plot, heatmap, and PCA plots. Ingenuity pathway analysis “IPA” was employed to identify the most significant canonical pathways and upstream regulators. Data were deposited (NCBI Gene Expression Omnibus accession series 235234).

**Supplemental figure legends:**

**Supplemental Fig 1:** Signaling responses to recombinant prorenin in brainstem neuronal cell cultures. A) Cells were treated with recombinant prorenin (0.1 μM) for 0, 30, and 60 minutes. Cell lysates were used for western blot analysis of phospho-p44/42 (pERK) and total ERK. pERK to total ERK ratio was calculated. Data were analyzed using one-way ANOVA followed by Tukey *post hoc* test. * p<0.005 vs t = 0 min, ** p< 0.001 vs t = 0 min. # p<0.005 vs t = 30 min. B) Cells were treated with either vehicle (control) or recombinant prorenin (1 μM) for 6 hours. Superoxide production was quantified using a luminometer in response to 1 mM NADPH. Data were analyzed by two-tail *t*-test. Data are expressed as mean ± SEM.

**Supplemental Fig 2:** Mean BP and activity evaluation upon PRR deletion in RVLM in females. A) Average hourly BP and activity at baseline. Shaded boxes depict dark cycle. B) Average daily BP and activity during DOCA-salt. C) Average daily BP and activity at baseline, and day 3 and day 13 during DOCA-salt. Data are expressed as mean ± SEM and were analyzed using repeated measures 2-way ANOVA followed by Sidak’s multiple comparison *post-hoc* test. *p<0.05 vs WT.

**Supplemental Fig. 3:** Pulse pressure and pulse wave velocity in WT and PRR^RVLM-Null^ females. A) Average daily pulse pressure (calculated by subtracting diastolic BP from systolic BP) at baseline and during DOCA-salt. B) Average pulse wave velocity at baseline and at day 13 on DOCA-salt. Data are expressed as mean ± SEM and were analyzed using repeated measures 2-way ANOVA followed by Sidak’s multiple comparison *post-hoc* test. ^¥^p<0.05 vs WT baseline and *p<0.05 vs PRR^RVLM-Null^ baseline.

**Supplemental Fig. 4:** A) Kidney, B) heart and C) spleen mass was measured for WT and PRR^RVLM-Null^ females. The relative organ mass was calculated as the organ weight divided by the body mass. Data are expressed as mean ± SEM.

**Supplemental Fig. 5:** Tubular sodium transporter evaluation. Complete blots from western blots assaying p-NCC, total NCC, NKCC2, p-NHE3, total NHE3 and αENAC. Ponceau red staining was used as a protein loading control.

**Supplemental Fig. 6:** Sodium balance in WT and PRR^RVLM-Null^ females. Mice were placed in metabolic cages for 24-hours sodium balance evaluation at baseline and at three different stages of DOCA-salt. Daily food intake, caloric intake, and feces weight are shown. The area under the curve (AUC) was calculated using the trapezoid method. Data are expressed as mean ± SEM.

**Supplemental Fig. 7:** Bulk RNA sequencing analysis in RVLM punches (n=3-4) from WT and PRR^RVLM-Null^ females. A) PCA analysis. B) Volcano plot. C) Heat map. D) Ingenuity pathway analysis: Top canonical pathways. E) Ingenuity pathway analysis: Top upstream regulators.

**References**

1. Zanaty M, Seara FAC, Nakagawa P, et al. β-Arrestin-Biased Agonist Targeting the Brain AT(1)R (Angiotensin II Type 1 Receptor) Increases Aversion to Saline and Lowers Blood Pressure in Deoxycorticosterone Acetate-Salt Hypertension. *Hypertension*. Feb 2021;77(2):420-431. doi:10.1161/hypertensionaha.120.15793

2. Nakagawa P, Gomez J, Lu KT, Grobe JL, Sigmund CD. Studies of salt and stress sensitivity on arterial pressure in renin-b deficient mice. *PLoS One*. 2021;16(7):e0250807. doi:10.1371/journal.pone.0250807

3. Shinohara K, Nakagawa P, Gomez J, et al. Selective Deletion of Renin-b in the Brain Alters Drinking and Metabolism. *Hypertension*. Nov 2017;70(5):990-997. doi:10.1161/hypertensionaha.117.09923

4. Fang S, Wu J, Reho JJ, et al. RhoBTB1 reverses established arterial stiffness in angiotensin II-induced hypertension by promoting actin depolymerization. *JCI Insight*. May 9 2022;7(9)doi:10.1172/jci.insight.158043

5. Segar JL, Balapattabi K, Reho JJ, Grobe CC, Burnett CML, Grobe JL. Quantification of body fluid compartmentalization by combined time-domain nuclear magnetic resonance and bioimpedance spectroscopy. *Am J Physiol Regul Integr Comp Physiol*. Jan 1 2021;320(1):R44-r54. doi:10.1152/ajpregu.00227.2020

6. Zhang J, Rudemiller NP, Patel MB, et al. Interleukin-1 Receptor Activation Potentiates Salt Reabsorption in Angiotensin II-Induced Hypertension via the NKCC2 Co-transporter in the Nephron. *Cell Metab*. Feb 9 2016;23(2):360-8. doi:10.1016/j.cmet.2015.11.013

7. Schreiber A, Shulhevich Y, Geraci S, et al. Transcutaneous measurement of renal function in conscious mice. *Am J Physiol Renal Physiol*. Sep 2012;303(5):F783-8. doi:10.1152/ajprenal.00279.2012

8. Veiras LC, McFarlin BE, Ralph DL, et al. Electrolyte and transporter responses to angiotensin II induced hypertension in female and male rats and mice. *Acta Physiol (Oxf)*. May 2020;229(1):e13448. doi:10.1111/apha.13448

9. Claflin KE, Sandgren JA, Lambertz AM, et al. Angiotensin AT1A receptors on leptin receptor-expressing cells control resting metabolism. *J Clin Invest*. Apr 3 2017;127(4):1414-1424. doi:10.1172/jci88641
