# Supplementary material for: Genetic Ablation of Prorenin Receptor in the Rostral Ventrolateral Medulla Influences Blood Pressure and Hydromineral Balance in Deoxycorticosterone-Salt Hypertension
Source: Function (Oxf). 2023 Aug 7;4(5):zqad043. doi: 10.1093/function/zqad043 (PMC10440998; doi:10.1093/function/zqad043)
Supplement: zqad043_Supplemental_Files [file zqad043_supplemental_files.zip › PRR-Manuscript-Suppl Figures_FINAL.pdf]

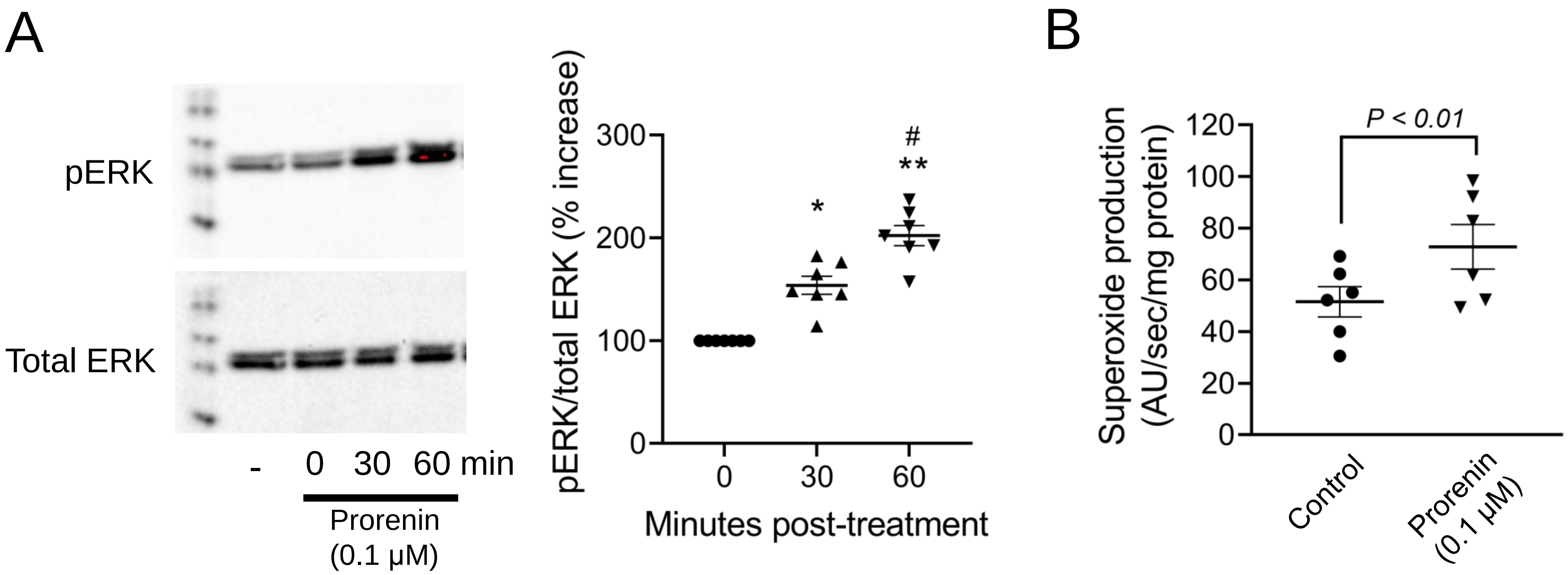
A
pERK
Total ERK
- 0 30 60 min
Prorenin
(0.1 μM)
pERK/total ERK (% increase)
300
200
100
0
*
**
#
0 30 60
Minutes post-treatment
B
Superoxide production
(AU/sec/mg protein)
120
100
80
60
40
20
0
P < 0.01
Control
Prorenin
(0.1 μM)

Fig. S1

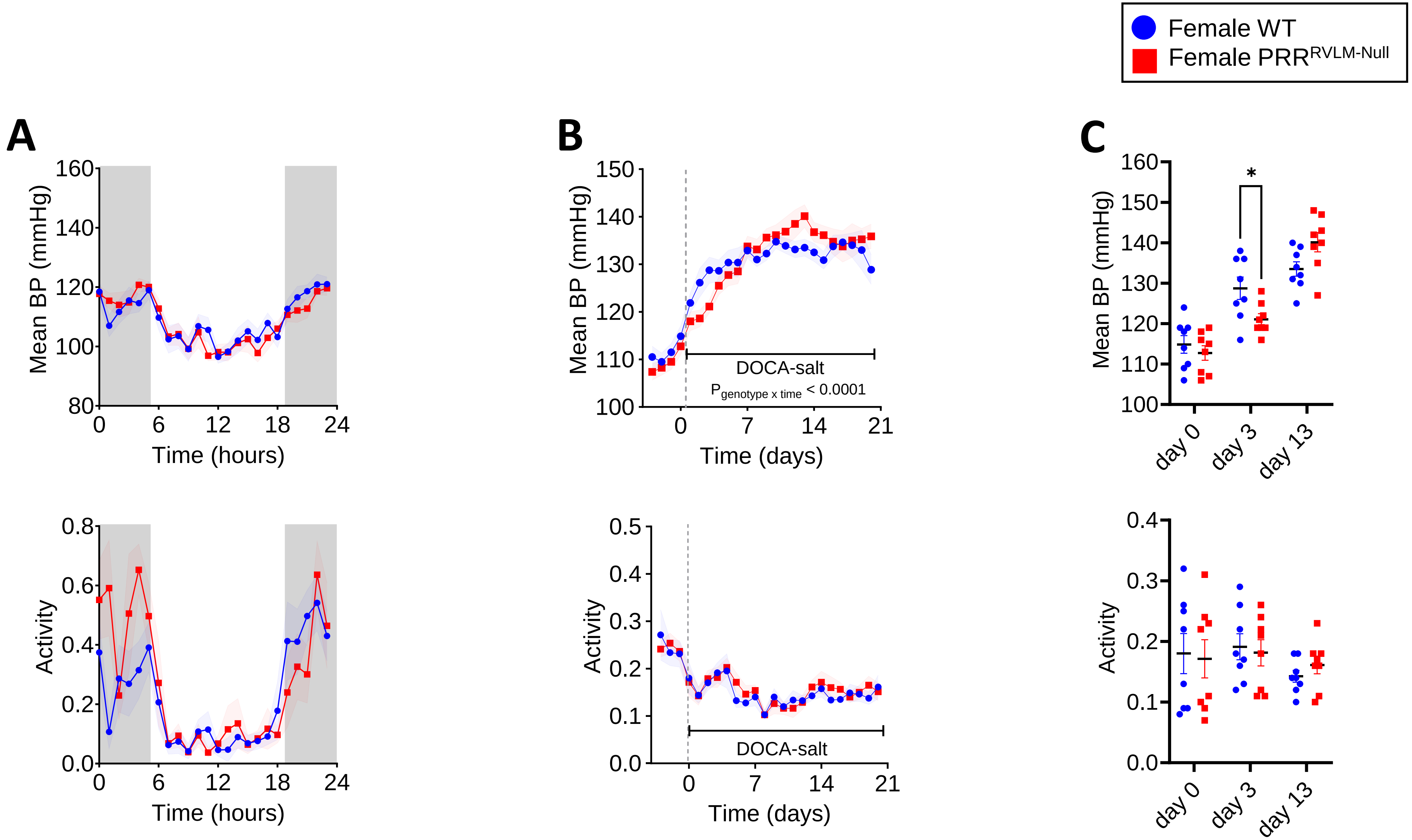

Female WT
Female PRR^RVLM-Null
A
B
C
Mean BP (mmHg)
Time (hours)
Activity
Time (days)
DOCA-salt
P genotype x time < 0.0001
*
day 0
day 3
day 13

Fig S2

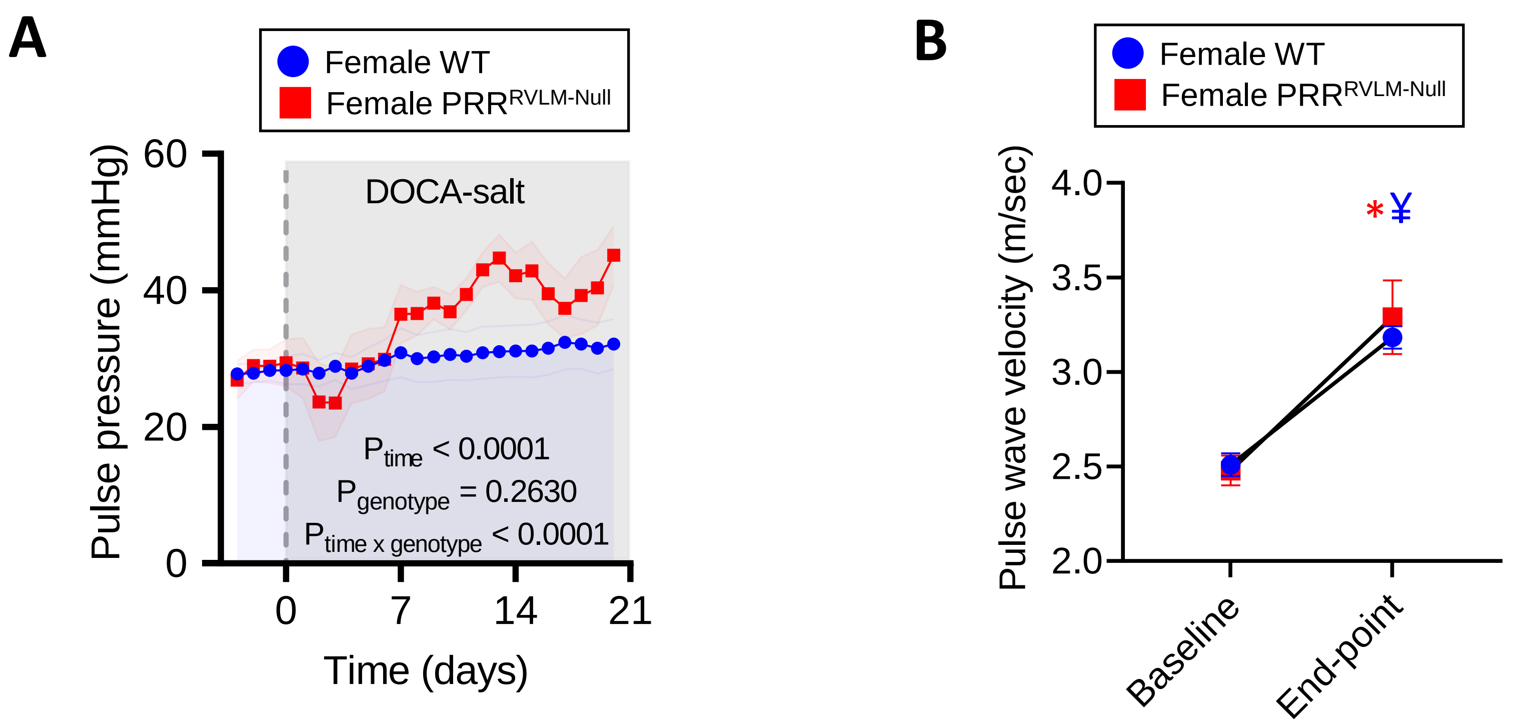
A
Female WT
Female PRR RVLM-Null
DOCA-salt
Pulse pressure (mmHg)
60
40
20
0
P time < 0.0001
P genotype = 0.2630
P time x genotype < 0.0001
0
7
14
21
Time (days)
B
Female WT
Female PRR RVLM-Null
Pulse wave velocity (m/sec)
4.0
3.5
3.0
2.5
2.0
* ¥
Baseline
End-point

Fig. S3

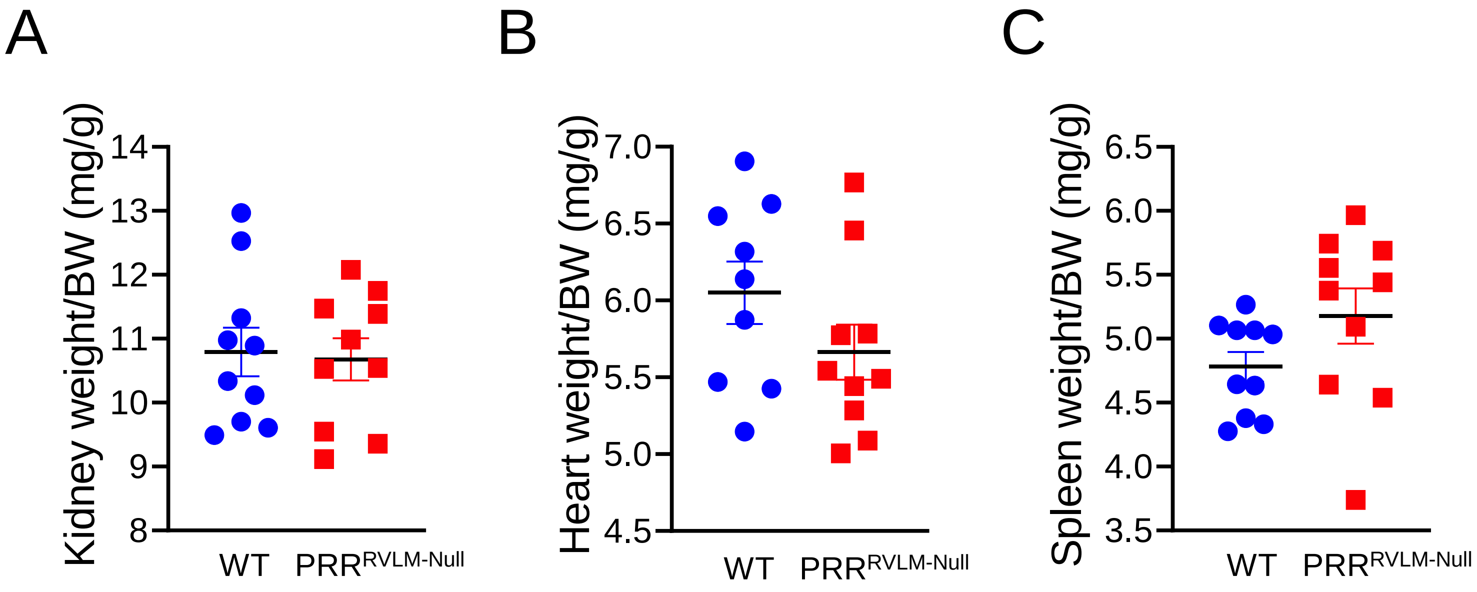
A
Kidney weight/BW (mg/g)
14
13
12
11
10
9
8
WT
PRR$^{RVLM\text{-}Null}$
B
Heart weight/BW (mg/g)
7.0
6.5
6.0
5.5
5.0
4.5
WT
PRR$^{RVLM\text{-}Null}$
C
Spleen weight/BW (mg/g)
6.5
6.0
5.5
5.0
4.5
4.0
3.5
WT
PRR$^{RVLM\text{-}Null}$

Fig. S4

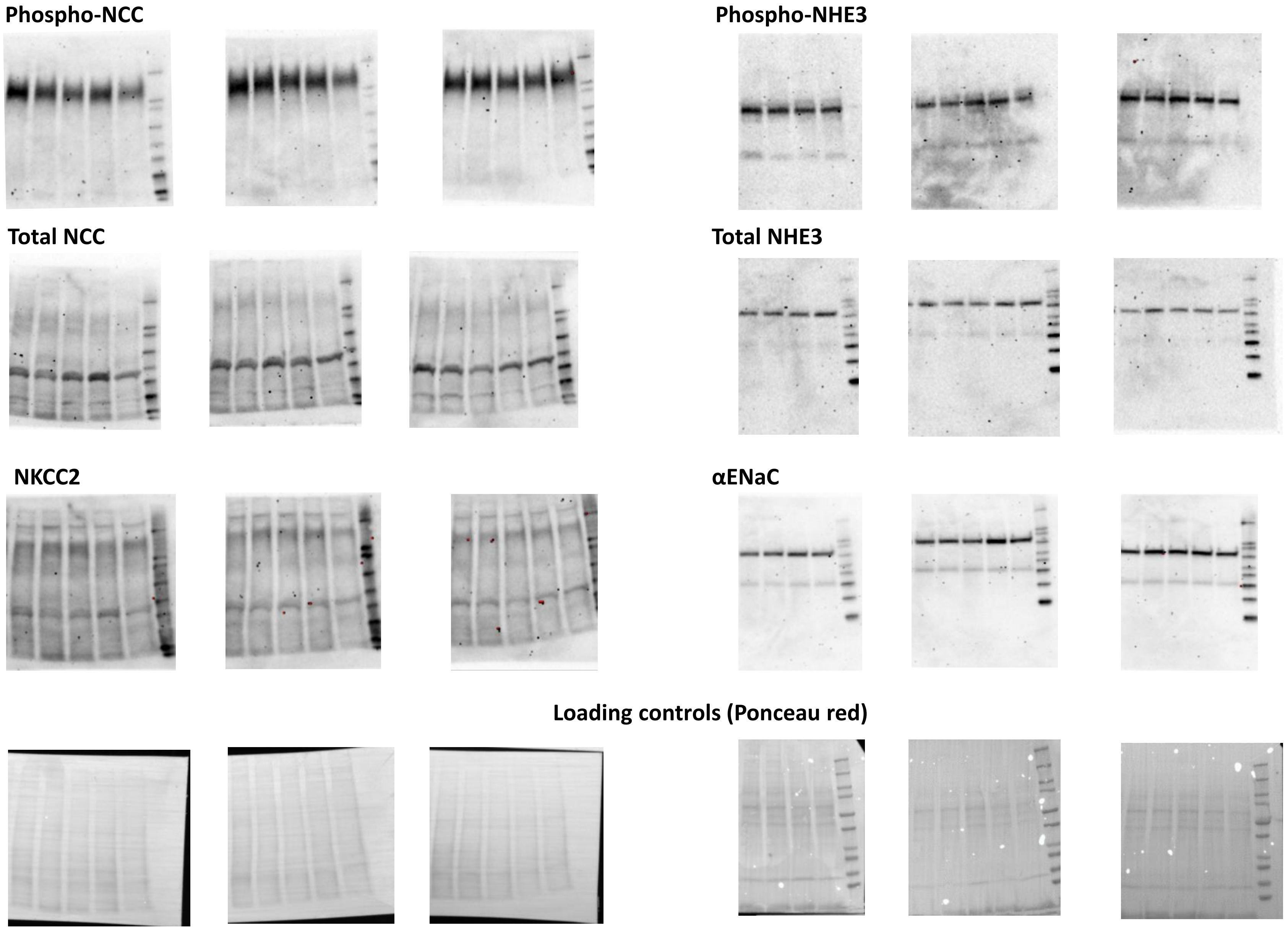
Phospho-NCC
Phospho-NHE3
Total NCC
Total NHE3
NKCC2
αENaC
Loading controls (Ponceau red)

Fig. S5

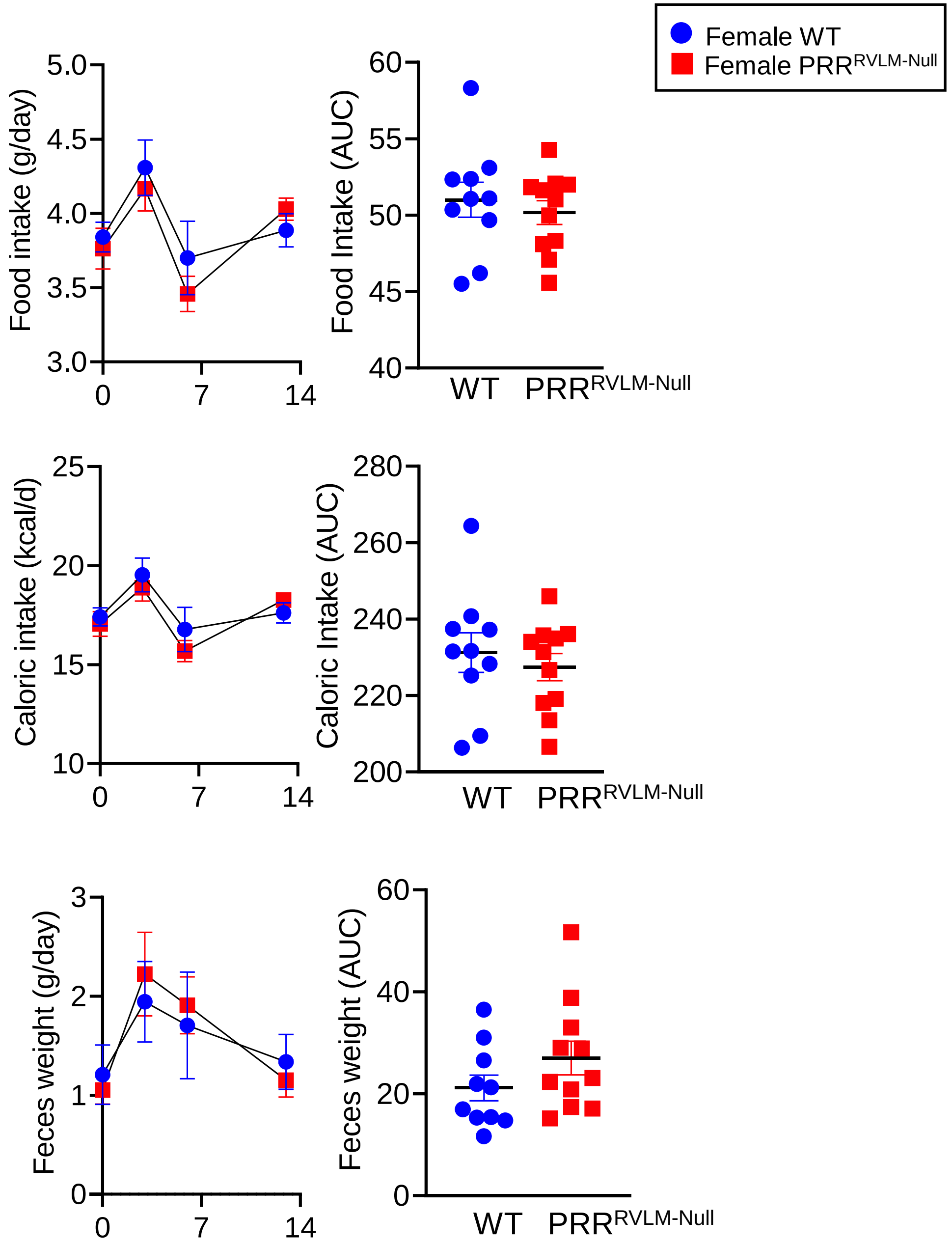
Female WT
Female PRR^RVLM-Null
Food intake (g/day)
5.0
4.5
4.0
3.5
3.0
0
7
14
Food Intake (AUC)
60
55
50
45
40
WT
PRR^RVLM-Null
Caloric intake (kcal/d)
25
20
15
10
0
7
14
Caloric Intake (AUC)
280
260
240
220
200
WT
PRR^RVLM-Null
Feces weight (g/day)
3
2
1
0
0
7
14
Feces weight (AUC)
60
40
20
0
WT
PRR^RVLM-Null

Fig. S6

A

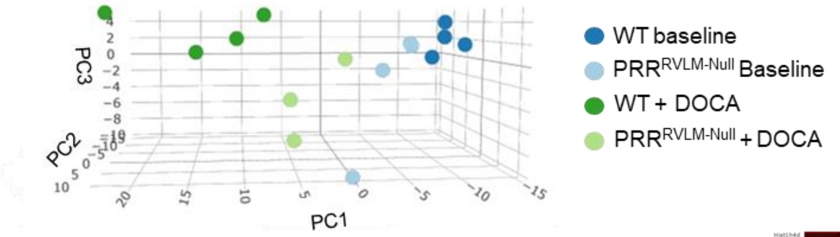

B

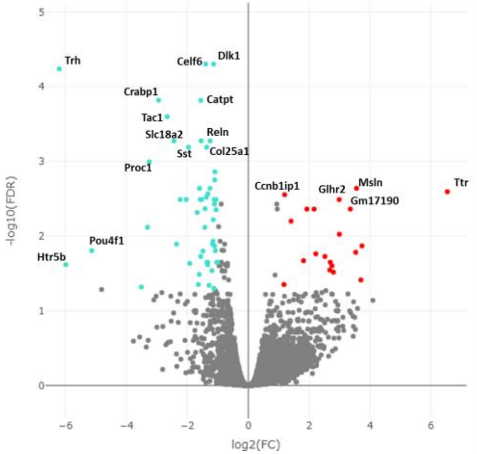

C

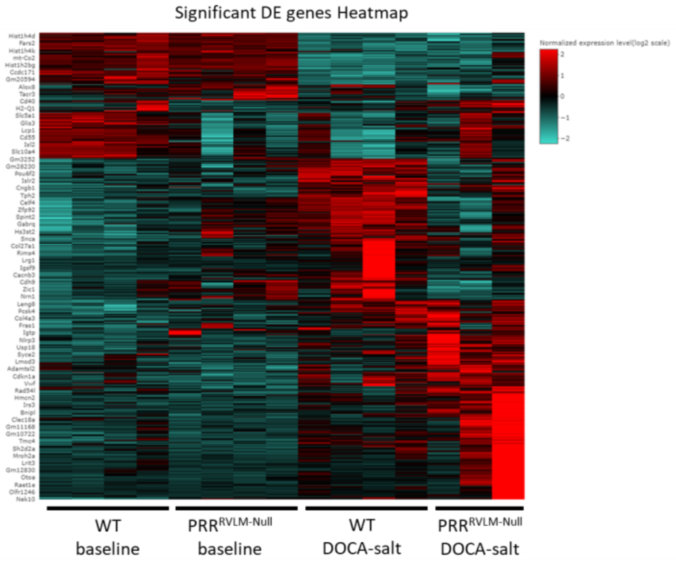

D

Top 5 canonical pathways	
Name	P-value
Transcriptional regulatory network in embryonic stem cells	1.61E-11
Hepatic fibrosis	1.06E-08
G-protein coupled receptor signaling	1.25E-07
DNA methylation and transcriptional repression signaling	8.03E-07
Phagosome formation	9.94E-07

E

Top 5 upstream regulators	
Name	P-value
POU4F1	7.56E-24
CREB1	1.09E-18
DAB3	5.02E-17
Actinonin	2.93E-16
Estradiol-17beta-benzoate	8.40E-15

Fig. S7
